# Transcaval Access and Closure Best Practices

**DOI:** 10.1016/j.jcin.2022.12.005

**Published:** 2023-02-27

**Authors:** Robert J. Lederman, Adam B. Greenbaum, Jaffar M. Khan, Christopher G. Bruce, Vasilis C. Babaliaros, Toby Rogers

**Affiliations:** a Cardiovascular Branch, Division of Intramural Research, National Heart, Lung, and Blood Institute, National Institutes of Health, Bethesda, Maryland, USA; b Structural Heart and Valve Center, Emory University Hospital, Atlanta, Georgia, USA; c St. Francis Hospital and Heart Center, Roslyn, New York, USA; d MedStar Washington Hospital Center, Washington, District of Columbia, USA.

**Keywords:** mechanical circulatory support, nonfemoral access, TAVR, TEVAR, transcaval, vascular access

## Abstract

Transcaval aortic access is a versatile electrosurgical technique for large-bore arterial access through the wall of the abdominal aorta from the adjoining inferior vena cava. Although counterintuitive, its relative safety derives from the recognition that interstitial hydraulic pressure exceeds venous pressure, so arterial bleeding harmlessly decompresses into the nearby caval venous hole. Transcaval access has been performed in thousands of patients for transcatheter aortic valve replacement and endovascular thoracic aneurysm repair and to avoid limb ischemia in percutaneous mechanical circulatory support. Transcaval access may have value compared with transaxillary or subclavian access and with surgical transcarotid access when standard transfemoral access is not optimal. The dissemination of transcaval access and closure techniques has been hampered by the unavailability of commercially marketed devices. This state-of-the-art review details exemplary transcaval technique, patient selection, computed tomographic planning, step-by-step access and closure, management of complications, and procedural troubleshooting in special situations. These contemporary best practices can help operators gain or maintain proficiency.

Transcaval access is an option for fully percutaneous introduction of large-bore devices to the aorta. It has been applied for transcatheter aortic valve replacement (TAVR),^1–6^ transcatheter endovascular aortic repair (TEVAR),^7^ transcatheter mechanical circulatory support (MCS) to avoid limb ischemia,^8,9^ and interventional pediatric cardiology.^10^

Transcaval access to the aorta entails delivering an introducer sheath from the femoral vein and inferior vena cava (IVC) across the retroperitoneal space into the adjoining infrarenal aorta (Central Illustration). Transcaval access to the aorta was first described in animals and in patients in 2013.^1,11^ The procedure was met with skepticism^12^ but ultimately demonstrated a favorable risk profile compared with other nonfemoral routes, including transaxillary or subclavian. ^6^ Transcaval access and closure entail procedural steps that may be unfamiliar to new operators. The purpose of this document is to elaborate steps and considerations to become proficient, on the basis of 9 years of the authors’ collective experience performing or proctoring more than 1,000 transcaval procedures and on the published data.^2–6^

## TRANSCATHETER ELECTROSURGERY PRINCIPLES FOR TRANSCAVAL ACCESS

Transcatheter electrosurgery is a versatile approach to traverse or cut tissue inside the blood pool.^13^ In contrast to electrocautery, which heats and coagulates tissue to stop bleeding, electrosurgery applies a high-frequency alternating current to vaporize tissue by heating. Ubiquitous via open surgical exposure, electrosurgery is now an essential endovascular tool.

Electrosurgery requires an electric circuit for current flow. Two electrodes connected to an electrosurgery generator create this electric circuit. Typically, the back end of the transcaval traversal guidewire is connected to a standard (Bovie) electrosurgery pencil. The other electrode is a large dispersive electrode (usually an adhesive electrolyte gel pad) attached to the patient’s skin. Through the principle of conservation of charge, equal current flows through the dispersive electrode and guidewire tip. Whereas the area of the dispersive electrode is about 20,000 to 40,000 mm^2^, the cross-sectional area of the guidewire is about 0.1 mm^2^. Current passes harmlessly through the skin and body but concentrates on the guidewire tip to vaporize and traverse tissue.

Tissue vaporization is enhanced by further concentrating current at the tissue contact point. We use microcatheters to insulate all but the guidewire tip and thereby concentrate current. In special situations (such as leaflet laceration), current is also concentrated by flooding the electrosurgical field with nonionic fluid such as dextrose-water or iodinated contrast to eliminate alternative current pathways through blood.

## ANATOMY AND PHYSIOLOGY OF TRANSCAVAL ACCESS AND COMPLICATIONS

Conventional wisdom that IVC or abdominal aortic perforation causes catastrophic bleeding^14^ derives from surgical experience with open or traumatized retroperitoneum. However, an intact retroperitoneal space behaves differently during transcatheter transcaval access. In fact, the transcaval approach is inspired by the observation that ruptured abdominal aortic aneurysms are hemodynamically tolerated when they spontaneously drain into the IVC. Similarly, traumatic femoral arteriovenous fistulae are often so well tolerated that they sometimes first manifest as high-output heart failure. In addition, iliofemoral veins are larger and more compliant than femoral arteries, are rarely obstructed, and can therefore accommodate larger introducer sheaths.

**FIGURE 1:**
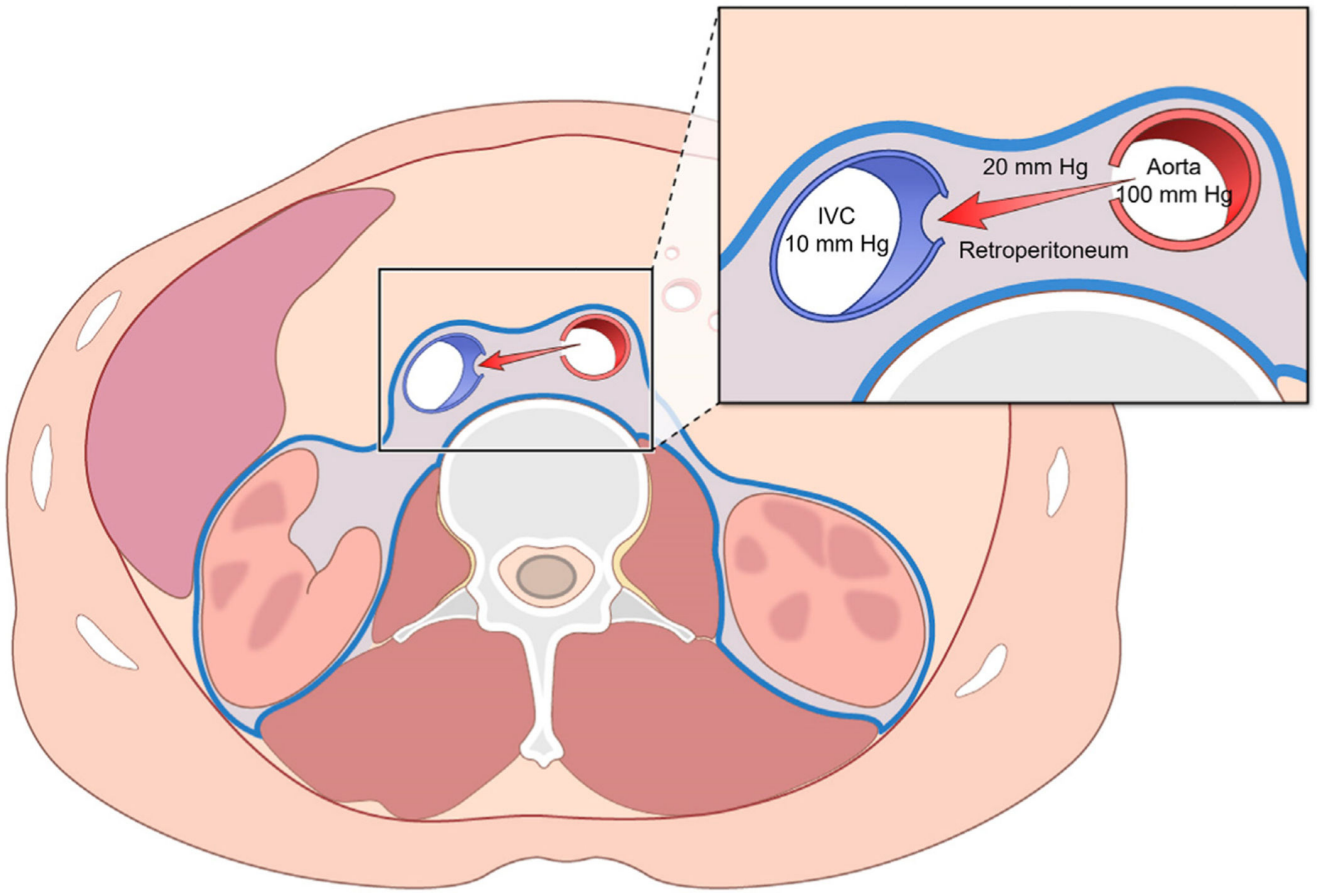
Physiology of Aortocaval Fistulae Retroperitoneal pressure is higher than venous pressure. Therefore, aortic bleeding shunts into the inferior vena cava (IVC).

The retroperitoneal space can be viewed as having 3 fluid compartments: aortic, venous, and interstitial. The hydrostatic pressure of the interstitial space is higher than the pressure in the caval vein. Consequently, adjoining holes in the aorta and cava shunt arterial blood across the pressurized interstitial space into the lowest pressure venous system rather than accumulating as retroperitoneal hemorrhage (Figure 1). In animals, intentional failure to close the aortocaval fistula is well tolerated and without retroperitoneal bleeding.^11^ Similarly, patients have tolerated temporary unrepaired aortocaval fistulae after inadvertent operator pull-through of nitinol occluder devices from the aorta to IVC.^2^

This physiology informs key elements of the transcaval procedure. First, isolated venous holes have little consequence, and there appears never to be a reason to evaluate for venous hemorrhage. Second, if ever there is even pinhole arterial bleeding, there must be a corresponding venous hole to decompress the arterial extravasation lest retroperitoneal hematoma accumulate. Specifically, transcaval introducer sheaths must never be allowed to exit the arterial lumen while still occluding the venous hole. Intentionally or inadvertently withdrawn transcaval introducer sheaths must always be further and fully withdrawn into the venous lumen. This was evident in the very first patient to undergo transcaval TAVR,^1^ who became hypotensive when the introducer sheath accidentally drifted outside the aorta but still occluded the cava; the blood pressure returned to normal immediately after the sheath was further withdrawn to allow aortic blood to return to the IVC.

## COMPUTED TOMOGRAPHIC PLANNING

Transcaval access should be planned on contrast-enhanced abdomen and pelvic computed tomographic (CT) imaging whenever possible, typically as part of the TAVR examination. Spatial resolution should be sufficiently high (≤1 mm isotropic) to minimize calcium blooming artifacts. The examination should span from celiac to common femoral arteries and be timed for contrast to opacify the aorta, IVC, and renal and iliofemoral arteries and veins.

Key objectives of transcaval planning on CT imaging are to identify a suitable target, to find lumbar vertebral and iliac artery landmarks to coregister the radiographic fluoroscopy and CT imaging, to define fluoroscopic projection angles to guide the procedure, and to plan bailout in case of complications.^15,16^

Table 1 summarizes CT planning considerations, including a checklist.

### TARGET SELECTION.

**FIGURE 2:**
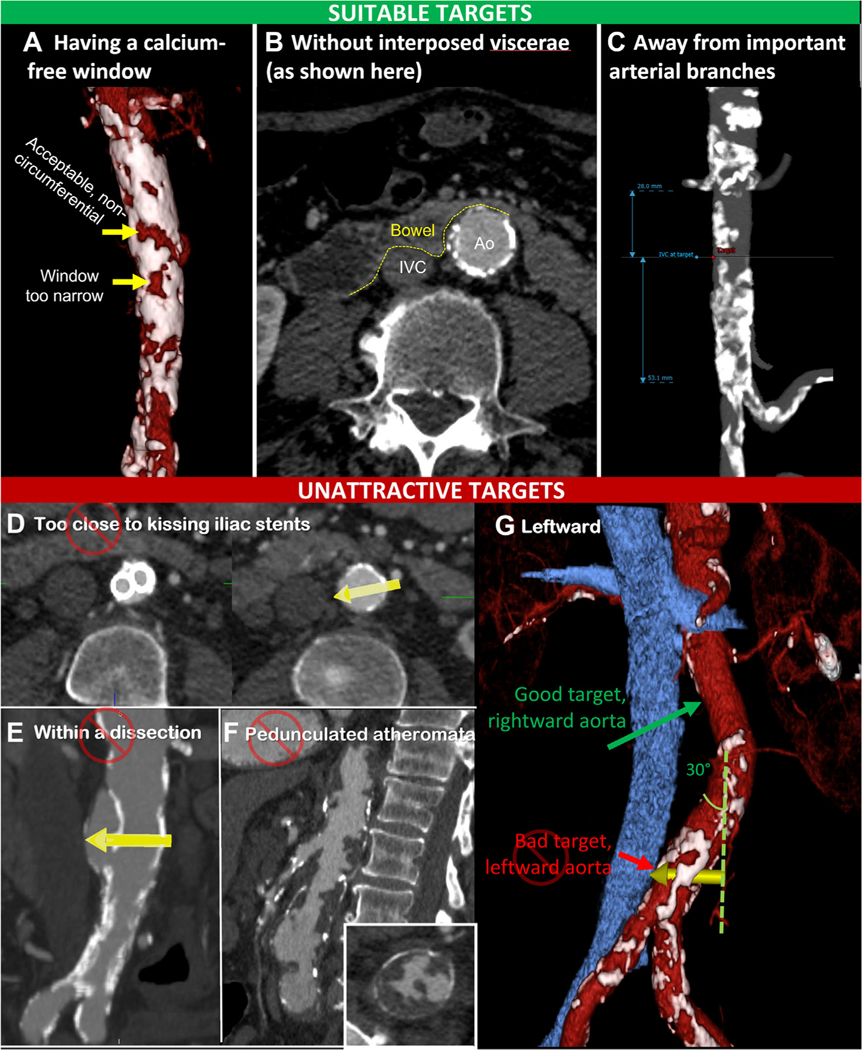
Target Selection Suitable targets are selected **(A)** having adequate noncircumferential calcium-free windows, **(B)** lacking interposed bowel as shown here, and **(C)** sufficiently away from important branches. Unsuitable targets include **(D)** targets in proximity to iliac stents that might interfere with device closure or bailout covered stent, **(E)** aortic dissection or intramural hematoma that might be disrupted or that might interfere with device closure, and **(F)** unattractive targets because of extensive risk for atheroembolism. **(G)** Leftward aortas make unsuitable targets, in contrast to rightward aortas. Ao = aorta; IVC = inferior vena cava.

An ideal transcaval target has a sufficiently large calcium-free window, without interposed bystandertissuesuch asbowel, sufficiently far from important branches to allow covered stent bailout. Figures 2A–2C depict these considerations.

#### Calcium.

Calcium is challenging to traverse with transcatheter electrosurgery. “Volume-rendered” CT imaging depicts calcium distribution and potential targets better than simply scrolling along axial slices. It is helpful to mark the aortic entry target on corresponding multiplanar CT reconstructions, as well as the corresponding IVC exit point.

The target needs to face the IVC directly across from it. Distance to the IVC appears unimportant for procedure success or complications.

If the target is surrounded circumferentially by calcification, then the calcium-free window should be >2 mm larger than the fully expanded TAVR introducer sheath. Importantly, even the nominally 14-F SAPIEN 3 eSheath (Edwards Lifesciences) transiently expands wider as the crimped valve is advanced through, so as a rule of thumb, the target should have a calcium-free window approximately 10 mm in at least 1 dimension.

In contrast, noncircumferential calcified transcaval targets reliably expand in an eccentric manner.

Within a given calcium-surrounded target “window,” we recommend entering the caudal aspect of the window as the traversal target, to ease advancement of introducer sheaths at an angle and to minimize interaction of the advancing sheath with calcium at the cephalad edge of the window.

#### Interposed structures.

Ensure no interposed viscera between the IVC and aortic targets, especially the duodenum, which can “drape” between the two. Expert operators occasionally choose trajectories that aim posteriorly to avoid partially interposed bowel. Interposed auxiliary renal arteries must be avoided, but venous plexi and lymphatic structures can be ignored.

#### Nearby arterial branches.

Targets need to be sufficiently far (approximately ≥10–15 mm) from important aortic branches to allow bailout covered stents a hemostatic landing zone seal without branch occlusion. Renal veins and lumbar arteries do not need special attention.

#### Mesenteric arteries.

Bailout covered stents will usually occlude the inferior mesenteric artery. We recommend checking the patency of the celiac and superior mesenteric arteries during planning, lest covered stent implantation cause mesenteric ischemia.

#### Distance from groin sufficient for the intended arterial introducer sheath.

The CT plan must ensure that the transcaval arterial introducer sheath will advance sufficiently deeply (≥3–5 cm) into the aorta for safe valve delivery and minimize risk that the sheath will inadvertently back out of the aorta during TAVR. Commonly selected sheaths have working lengths of 30, 33, 35, and 40 cm; 65-cm sheaths may be too long to use with closure-device delivery assemblies. We approximate the required sheath working length by 1) measuring a straight line from the bottom of the femoral head (to compensate for skin depth and for inadvertently low venous puncture) to the corresponding caval exit point; 2) adding the caval-aortic distance; and 3) adding 5 cm to ensure adequate sheath “purchase” inside the aorta.

#### Aortic aneurysm, dissection, atheroma, grafts, and stents.

A common misconception is that aortic aneurysm contraindicates transcaval access. In practice, aneurysmal or ectatic aortic pouches are attractive targets. Indeed, lamellated thrombus appears to facilitate early hemostasis of nitinol occluder devices. In selecting targets, we prefer the nitinol occluder disc to seat snugly within the diseased aortic wall or pouch. We are not aware of early or late complication of such approaches. The chief limitation is the availability of sufficiently large bailout covered stent devices and suitable proximal and distal “necks.”

In contrast, aortic dissection above the intended target is a relative contraindication to transcaval access. Operators should consider 1) the risk for occluder device entrapment by the dissected tissue during withdrawal, expansion, and implantation; and 2) how the transcaval introducer sheath might interact with or exacerbate dissections.

Pedunculated atheromata are another relative contraindication to transcaval access. Intra-aortic manipulation and expansion of nitinol occluder devices in this setting risks distal atheroembolism. Figures 2D–2F depict examples of unattractive targets such as dissection.

Transcaval access is attainable across fabric surgical grafts.^17,18^ Perigraft fibrous adhesions may reduce the likelihood of retroperitoneal bleeding in this setting. We are not aware of transcaval access across endovascular aortic repair devices.

Aortoiliac stents, solitary or “kissing,” may protrude into the lower aorta and interfere with closure device or bailout covered stent deployment. Targets should therefore be sufficiently far from iliac stents.

#### Leftward and tortuous aorta.

Leftward-leaning aortas make transcaval procedures more difficult to execute. Introducer sheaths tend to “skyve” the aortic wall, risking dissection, and closure devices become difficult to deploy at acute angles. We consider aortic segments leaning approximately ≥30° leftward from vertical to be unattractive targets.

Aortic tortuosity is common among transcaval candidates. In addition to considering the “leftwardness” of targets, we advise operators to be cognizant of 2 considerations. First, the left-right relationship of the IVC to the aorta varies along the course of tortuous aortas; specific projection angles should be selected for each individual target in such patients. Second, operators should consider the possible injurious interaction of bulky closure devices with the wall of tortuous aortas during closure maneuvers.

### SELECT PROJECTION ANGLES FOR THE TRANSCAVAL PROCEDURE.

Fluoroscopic projection angles should be tangential to the aorta at the selected target, at the point closest to the IVC. A conjugate orthogonal projection (90° oblique to the first) is important to ensure appropriate en face (“lateral”) aim.

Projection angles do not need craniocaudal angulation, which means that they are not necessarily orthogonal to the long-axis aortic centreline automatically provided by popular CT analysis software. Some operators disagree.

**FIGURE 3:**
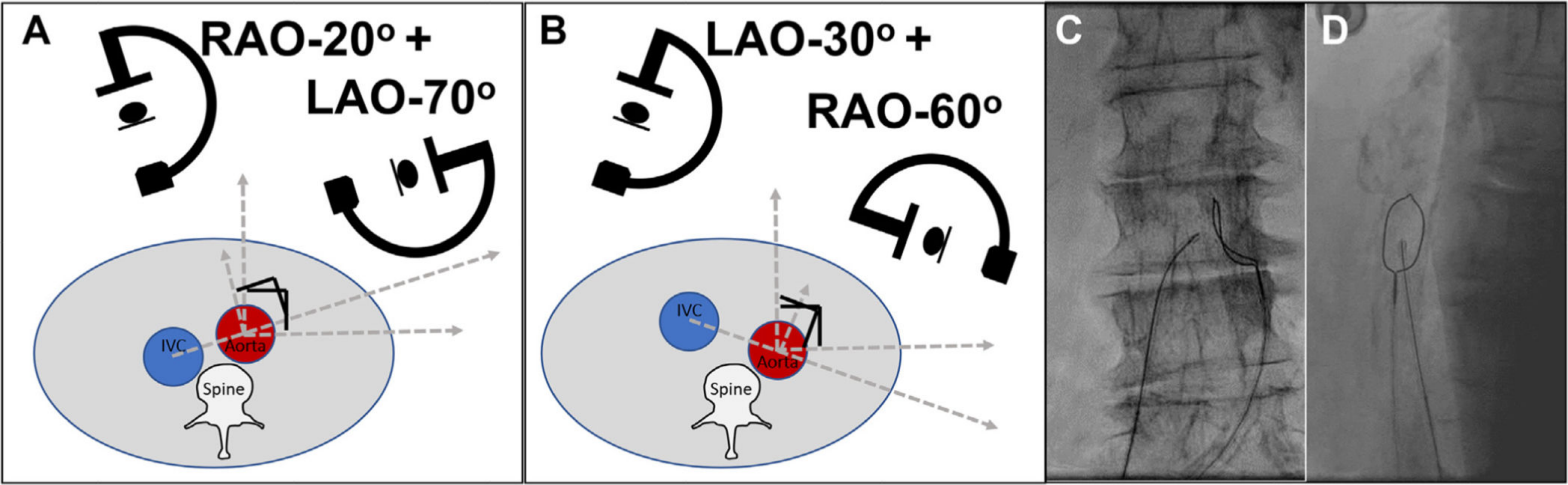
Projection Angles **(A)** Typical cross-sectional relationship of inferior vena cava (IVC), aorta, and spine. Vessel center points define frontal right anterior oblique (RAO) and conjugate en face left anterior oblique (LAO) projections. **(B)** Less common aorta/IVC location, with attendant LAO frontal and conjugate RAO en face projections. **(C,D)** Representative frontal **(C)** and en face **(D)** projections.

The angles are most easily determined by connecting the centers of the IVC and aorta on an axial multiplanar reconstruction, through the aortic target. The frontal projection is the angle of that centerline with regard to an anteroposterior line. The lateral en face projection is 90° perpendicular to the frontal (Figure 3).

### VISUALLY COREGISTER CT IMAGING WITH RADIOGRAPHIC FLUOROSCOPY.

The CT plan must correspond unambiguously with radiographic fluoroscopy for safe transcaval access and closure. We recommend using bony landmarks (lumbar vertebrae and iliac crests) corroborated by angiographic landmarks (aortoiliac bifurcation and/or renal artery or renal parenchyma). It is helpful to depict these landmarks on computed tomography–simulated fluoroscopic images that can be displayed to the operator during the procedure.

**FIGURE 4:**
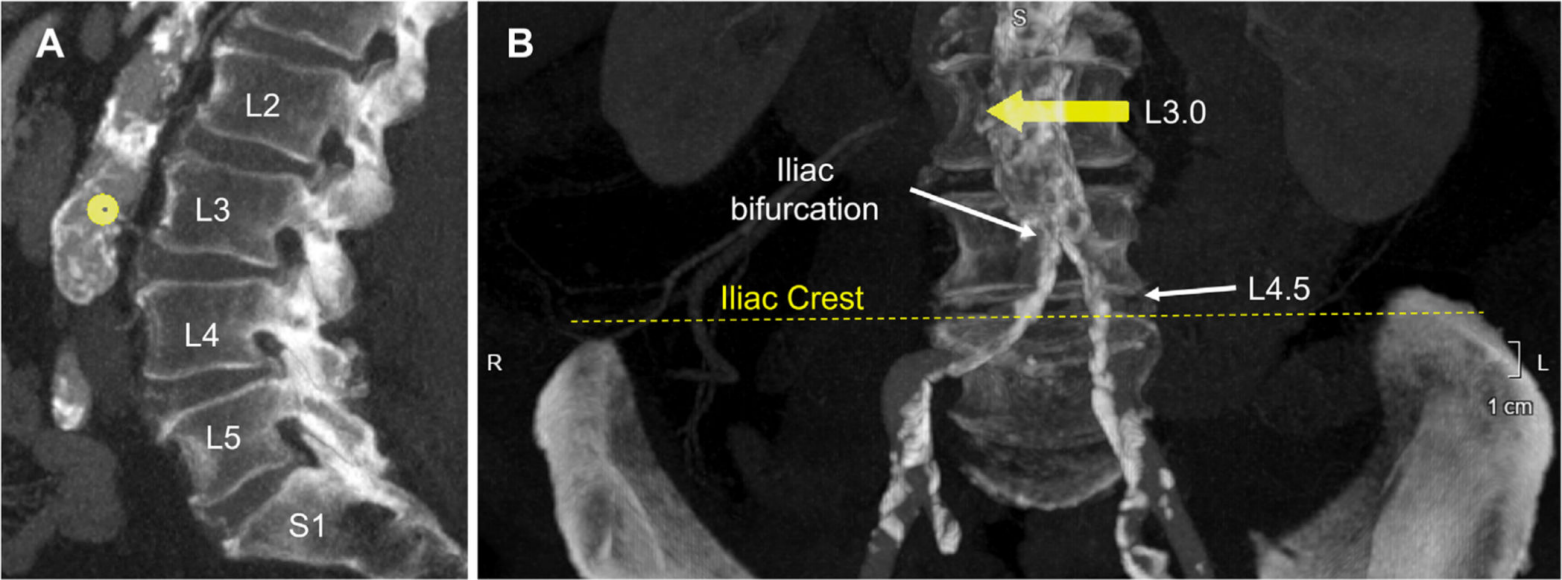
Landmarks Landmarks to coregister computed tomography–based traversal targets with fluoroscopy. **(A)** Lumbar vertebrae on sagittal reconstruction. The trapezoidal S1 sacral vertebra is distinguished from the rectangular L5 vertebra. **(B)** Iliac crests correspond with L4-L5 vertebral interspace and aortoiliac bifurcation. This target overlies the mid L3 (L3.0) vertebra.

Lumbar vertebrae should be identified on lateral multiplanar CT reconstruction, where the rectangular fifth lumbar vertebra (L5) is distinguished from the angulated and triangular first sacral vertebra (S1) (Figure 4).

The L4-L5 interspace typically coincides with 1) the top of the iliac crests; and 2) the aortoiliac artery bifurcation in a straight anteroposterior projection. The 2 determinations combined should corroborate each other.

Identify the target. Many operators describe lumbar location numerically: L3.0 is the middle of the L3 vertebral body and L3.5 the middle of the L3-L4 interspace. Lumbar vertebrae should not be identified by counting downward from thoracic vertebrae, because “false” ribs are common confounders. Instead, planner should “count upward” from the L5-S1 interspace identified on a sagittal CT scan.

### BAILOUT BALLOON TAMPONADE AND COVERED STENT PLAN.

The CT plan must identify the diameter of a potential balloon for aortic tamponade. We typically use compliant balloons sized 120% to the aorta at the level of the traversal. The objective of balloon tamponade is to occlude flow and allow the nitinol occluder time to thrombose and not to perform high-pressure angioplasty.

The CT plan must identify the diameter and length of a potential covered stent bailout, along with a tolerable femoral artery access site. This usually requires identifying a specific bailout device. Transfemoral artery bailout with a large-diameter stent delivery system undermines the rationale for transcaval TAVR; because it is required so infrequently, the risk may be considered acceptable.

Several large-diameter covered stents are commercially available, but most require excessively large delivery systems or exhibit excessive through-graft flow and fail to achieve immediate hemostasis (ie, type IV endoleak through excessive porosity; see “Remedies”).

This presupposes an available iliofemoral artery with known, if limited, diameter to allow bailout access. We suggest that the CT plan include a specific bailout covered stent device selection and recommended (right vs left) access route.

## PERFORMING TRANSCAVAL ACCESS AND CLOSURE

### EQUIPMENT.

Both routine and bailout equipment should be assembled before undertaking the procedure. Table 2 lists mandatory and optional equipment recommendations.

Bailout equipment (balloon aortic tamponade catheters, suitable introducer sheaths, and suitable covered stents) must be gathered in the procedure room, to ensure ready availability.

### PATIENT PREPARATION.

Table 3 suggests staff briefing topics for centers that use transcaval access infrequently.

Transcaval procedures can be performed using moderate sedation or general anesthesia as desired. Among awake patients, extra sedation or analgesia should be administered immediately before electrosurgical traversal.

Before the patient is draped, the electrosurgical dispersive electrode should be applied evenly to an area of clean and dry skin (typically on the thigh) to reduce the risk for skin burns and ensure effective electrosurgical traversal. Teams should be prepared temporarily to elevate the arms of draped patients to facilitate lateral fluoroscopy in large patients when needed.

The electrosurgery pencil should be connected to the monopolar terminals on the electrosurgery generator, set to “pure” cutting mode starting at 30 to 50 W. “Coagulation” or “cautery” mode should be disabled (0–1 W).

Some electrosurgical pencils have clear polymer coating on the flat faces (such as Medtronic Edge coating). These preclude effective guidewire electrosurgery. Ensure that uncoated stainless-steel pencils are used; alternatively, insulation can be debrided with a scalpel as a last resort.

### BEGINNING THE PROCEDURE.

The objective is to create pinhole across the aortic wall to deliver a 0.014-inch guidewire and then progressively to enlarge the hole until the transcaval TAVR introducer sheath is in place.

The right femoral vein is preferred over the left, to improve transaortic sheath advancement against possible resistance. The femoral vein should be accessed as cephalad as possible to shorten the skin-to-aorta distance in cases of borderline sheath length. Suture-mediated closure devices (“preclosure”), “figure-of-8” suture, or a combination can be used as per local practice for large-bore venous closure.

The larger femoral artery should be accessed to allow aortography, to position a snare target for transcaval guidewire traversal and ensnarement, and for bailout covered stent implantation.

Next, the fluoroscopy and features from the baseline CT scan should visually be coregistered (in the mind of the operators), using anatomical fiducial landmarks such as the iliac crests to align specific lumbar spine segments. Adjusting the table height to position the target aorta at the isocenter (the axis of C-arm rotation) eases subsequent procedural steps.

A pigtail aortography catheter should be positioned below the renal arteries, and the transvenous guiding catheter should grossly be positioned at the traversal target, using a wide (~32 cm) fluoroscopic field of view. The C-arm should be rotated to the computed tomography–predicted frontal projection angle. Low-volume aortography (5–10 mL over 1 second) is usually adequate. Many operators prefer digital subtraction for later comparison with postclosure aortograms; unsubtracted images are helpful to depict aortogram bony landmarks during traversal. Experience has revealed little or no value to baseline (or completion) vena cava angiography, which we no longer perform.

We recommend full heparin anticoagulation to achieve an activated clotting time >250 seconds before traversal. Heparin suppresses intravascular thrombosis induced by guidewire electrosurgery or on the target snare that will be positioned in the aorta.

#### The snare and crossing system.

The crossing system ultimately positions a rigid 0.035-inch guidewire into the aorta from the femoral vein. Crossings should be orthogonal to the aorta to ease device advancement and to reduce aortic injury; more oblique, cephalad trajectories are undesirable.

We prefer rigid and exchangeable 0.014 inch × 300 cm guidewires for electrosurgical traversal. In our early experience we used a chronic total occlusion guidewire (Confianza Pro 12, Asahi) with the distal 11 mm manually amputated to confer extra stiffness. More recently we use an unmodified stiff guidewire (Astato XS 20, Asahi).

The guidewire is loaded into a coaxial system composed of a 0.014-inch microcatheter inside a 0.035-inch microcatheter; the 0.014-inch microcatheter must be longer than the 0.035-inch microcatheter, which in turn must be longer than the guiding catheter system. We prefer a locking hub–less 0.014-inch marker-tipped 0.014-inch microcatheter (145-cm PiggyBack, Teleflex), although most 0.014-inch microcatheter alternatives suffice. The guidewire and 0.014-inch microcatheter are loaded inside a shorter (90-cm) 0.035-inch braided microcatheter, preferably with lubricious coating (such as the 0.035-inch NaviCross, Terumo).

**FIGURE 5:**
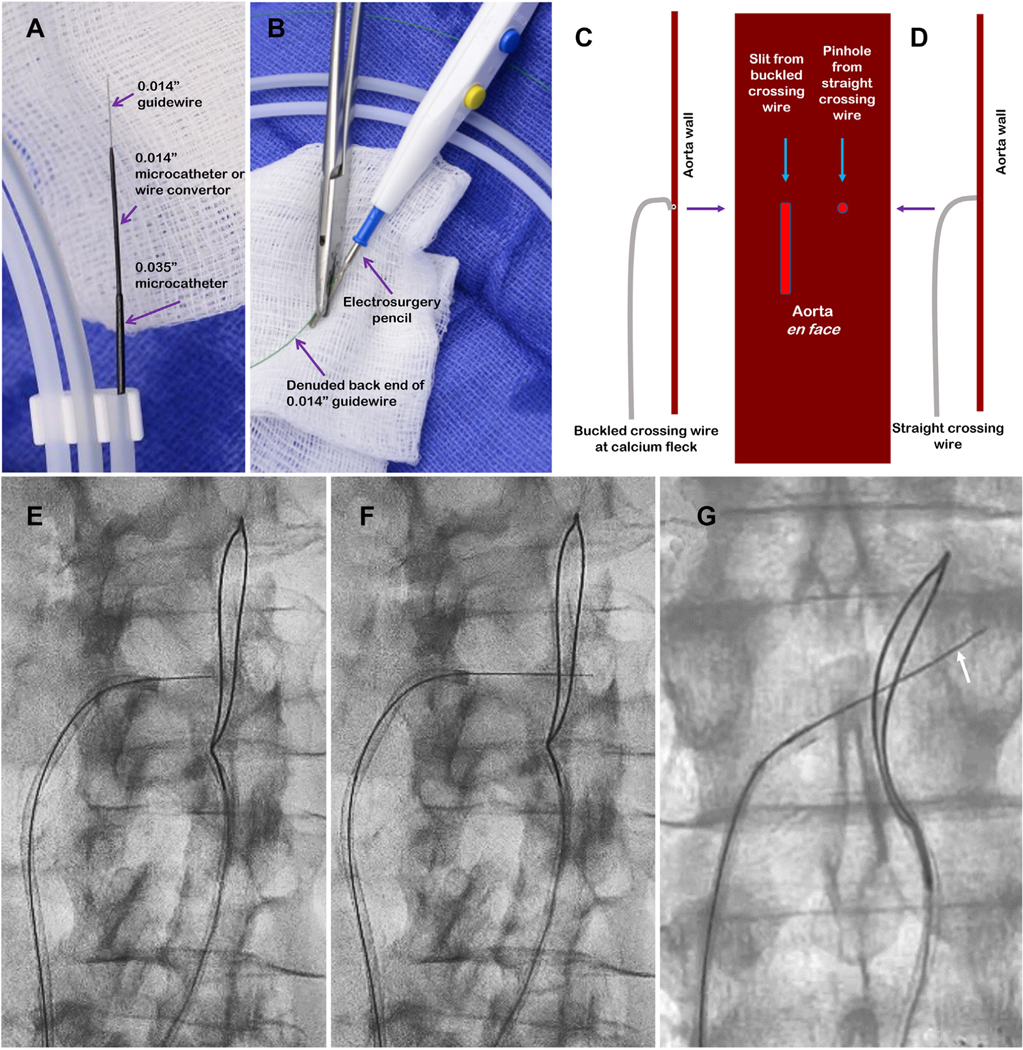
Crossing Traversal equipment and strategy. **(A)** A 0.014-inch guidewire within a (~135-cm) 0.014-inch microcatheter, within a (~90-cm) 0.035-inch microcatheter. **(B)** Guidewire back-end insulation is denuded and clamped to a bare electrosurgery pencil. **(C)** Electrified guidewires buckle against mural calcium. If electrified once buckled, advancement can create a lengthy slit in the aortic wall that may cause extravasation. **(D)** Properly oriented guidewires, when electrified, generate pinholes that recoil. **(E,F)** “Two-step” electrosurgical traversal. **(E)** Electrified wire exits the inferior vena cava and is then advanced along with the microcatheter without electrification until it abuts the aorta. **(F)** The wire is electrified a second time across the aortic wall and the snare. Electrified wires should cross “like buttah” and should not buckle. **(G)** Without electrification, the wire is further advanced until it subtly buckles **(arrow)** against the contralateral aortic wall, confirming intraluminal position.

The guiding catheter, typically renal length about 55 cm internal mammary or short renal double curve 1, is selected to point horizontally toward the target aortic wall. The final crossing system consists of the guidewire inside a 0.014-inch microcatheter inside a 0.035-inch microcatheter inside a rotating hemostatic adapter on a 6- to 8-F guiding catheter (Figures 5A and 5B). Some operators substitute a deflectable guiding sheath (eg, Nagare, Terumo) for these preshaped guiding catheters, especially because they will be used during later closure steps, in which case longer microcatheters must be employed.

A single-loop snare (Amplatz Goose Neck [Medtronic] or ONE Snare [Merit]), with a diameter 5 to 10 mm larger than the aorta, is loaded into a 6-F guiding catheter (typically a JR4) with a rotating hemostatic adaptor and with the snare lock loaded for convenience. The snare is initially placed cephalad to the target and slowly withdrawn while torquing until it drapes along the right wall of the aorta at the target. In the computed tomography–selected frontal projection angle, the properly positioned snare loop should appear like a crescent moon (Figures 3C and 3D).

#### Readying for crossing.

The back-end 1 cm of the 0.014-inch guidewire is stripped of insulation using a scalpel to enable electric conduction and can be clamped to the electrosurgical pencil using metallic forceps.

At this point, the traversal guiding catheter is finely positioned toward the target by alternating between frontal and orthogonal en face projections, while torquing to aim at the snare “bull’s-eye” in the lateral en face projection. Take care that these projections are truly 90° apart. In the typical configuration (IVC to right of aorta, catheter pointed from IVC to aorta), clockwise rotation of the caval guiding catheter aims posteriorly and counterclockwise rotation aims anteriorly. Further fine positioning takes into account individual pathology predicted from the CT plan, such as anterior bowel or focal anterior calcification.

When there is a narrow calcium-free window on the aorta, we recommend crossing at the most caudal aspect of the target, because large introducer sheaths meet resistance on cephalad aspects of such windows.

We recommend multiple alternating frontal and lateral fine-tuning views to ensure accurate trajectory planning. It is helpful for the first operator to retain guiding catheter position carefully until traversal is successful.

Try to avoid inadvertently engaging a renal or lumbar vein with the guiding catheter. This is evident on visual-tactile feedback and on lateral en face fluoroscopy.

Ensure that there are no loops or wet towels that might cause an electric “short circuit.” Ensure that the microcatheter is unlocked to allow free guidewire advancement. With the guiding catheter pointed at the target, the guidewire touching the wall of the cava, and the microcatheter within a few millimeters of the tip, the operator is ready for electrosurgical traversal.

#### Electrosurgical crossing.

Table 4 lists key teaching points for crossing.

Cutting mode is usually activated by the yellow-colored switch on electrosurgical pencils (or a foot pedal).

Electrification must be deliberate and brief. Electrified guidewires should be advanced immediately and steadily at a pace of about 2 mm/s for no more than about 2 to 3 seconds at a time. Subtle buckling of the electrified guidewire suggests deflection by calcium and requires repositioning toward a calcium-free window. More than subtle buckling causes undesirable tissue slicing rather than pinholes (Figures 5C and 5D), so electrification should be stopped immediately. Electrification should also be stopped immediately as soon as the guidewire enters the target aorta, as demarcated by angiography and by the prepositioned snare. Electrification cumulatively carbonizes the guidewire tip, reducing electrosurgical efficacy and resulting in tissue heating rather than vaporization.

When the IVC and aorta are more than 5 to 10 mm apart, it can be helpful to use 2-step electrosurgical traversal, first to exit the cava and second to enter the aorta. In these cases, it is helpful to check the lateral en face projection between the first and second electrifications, to ensure that the trajectory remains as intended.

When the traversal guidewire enters the aorta, it should be advanced without additional electrification until subtle buckling is evident against the contralateral luminal wall. This confirms the wire has entered the aortic lumen (Figures 5E–5G).

#### Troubleshooting electrosurgical wire crossing.

Failure to advance the electrified guidewire without buckling or deflection represents 1 of 2 problems: calcium or electric failure.

Aortic wall calcification in general cannot be traversed by transcatheter electrosurgery. Fortunately, subtle repositioning of the guidewire trajectory usually finds a crossable path.

Absent a calcific obstacle, electric failure is the most likely culprit. Common possibilities are listed in Table 5.

One quick way to test a functional electrosurgery circuit is to stack wet gauze on the patient’s bare skin and briefly touch the electrified guidewire tip to the gauze; scalding indicates a functioning circuit.

Finally, operators should be aware that newer electrosurgery generators incorporate adaptive circuitry that automatically changes voltage or current in response to guidewire char-associated impedance. These can cause unpredictable results ranging from electrosurgery failure to melting the guidewire solder in vivo. We find the oldest, least sophisticated generator (Valleylab Force FX/A, Medtronic) to be the most dependable.

#### Countertraction and exchange for a rigid 0.035-inch guidewire.

**FIGURE 6:**
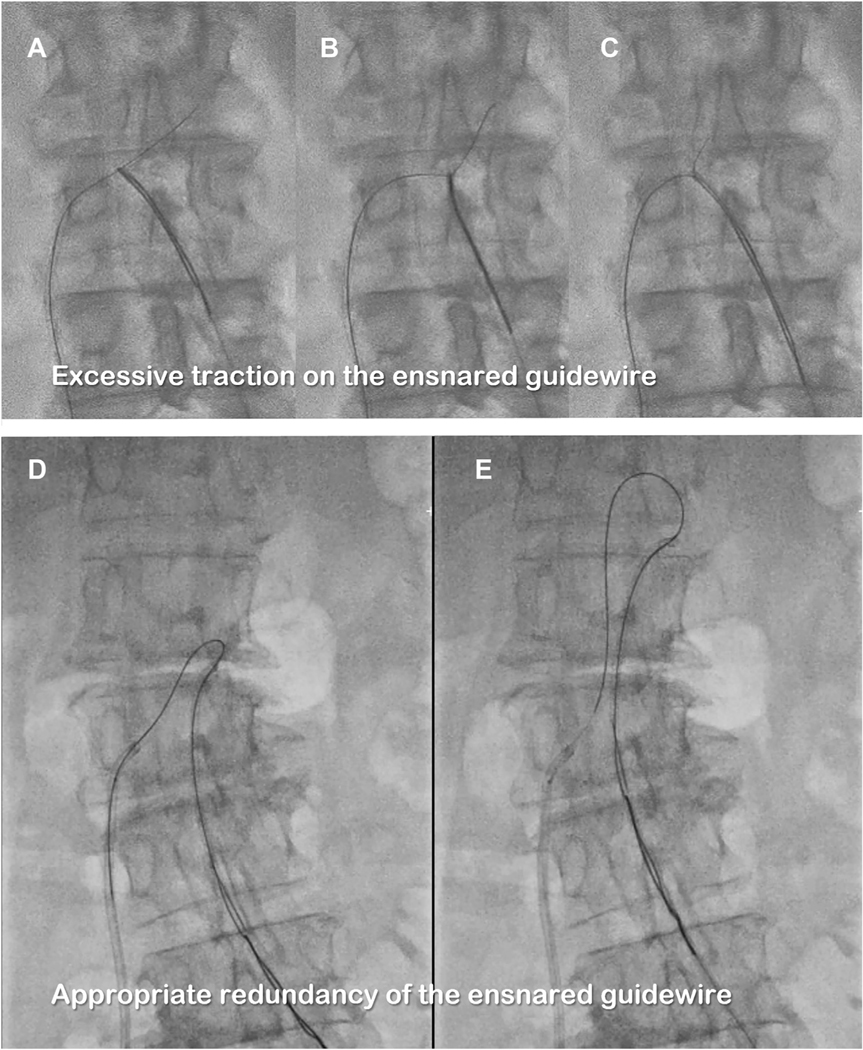
Avoid Sucking Snare Guide Out of Aorta Inadvertently During Wire Ensnarement **(A-C)** Excessive traction on the ensnared guidewire may pull the guiding catheter outwards **(C)**, risking aortic perforation. **(D)** If the guidewire is fed toward the aorta during snare invagination, the guiding catheter does not jump. **(E)** Redundant guidewire safety loops avoid aortic injury during advancement.

The guidewire should be ensnared and the guidewire advanced. The softer distal guidewire (1 to 2 cm) eases ensnarement. Special care is required to avoid inadvertently pulling the transfemoral aortic guiding catheter through the transcaval hole and out of the aorta or slicing the aortic wall with excessive traction on the ensnared guidewire. Instead, redundant guidewire is advanced into the aorta from the IVC, while simultaneously torque-advancing the aortic guiding catheter, before invaginating the guidewire into the aortic guiding catheter (Figure 6). The transfemoral aortic guiding catheter is advanced to the level of the aortic arch while feeding the transcaval guidewire antegrade.

With countertraction applied between the caval and aortic guiding catheters to straighten the guidewire, the 0.014-inch microcatheter is advanced across the transcaval tract to the thoracic aorta. In case of failure to advance, the 0.014-inch microcatheter and 0.035-inch microcatheters are exchanged for a noncompliant balloon dilatation catheter 2.0 to 4.0 mm in diameter. Full balloon expansion should be achieved across both aortic wall and caval walls. Such “small-balloon” predilatation, with the transcaval guidewire tethering open the aortocaval fistula, tends not to cause bleeding or hypotension.

Over the 0.014-inch microcatheter, the 0.035-inch microcatheter is next advanced deeply into the aorta. The 0.014-inch guidewire is the released from the snare. The transfemoral snare catheter is exchanged for the pigtail aortography catheter used for TAVR and later for transcaval tract closure.

The rigid 0.035-inch guidewire (single-curve Lunderquist Extra-Stiff, Cook) is advanced through the 0.035-inch microcatheter under fluoroscopic visualization (to ensure that the microcatheter does not prolapse out of the aorta into the cava) and parked just distal to the aortic arch.

#### Transcaval introducer sheaths.

With the Lunderquist guidewire in place, the 0.035-inch microcatheter is withdrawn. The TAVR introducer sheath assembly (or the isolated dilator if using an expandable introducer sheath, eg, the eSheath) is advanced smoothly from the IVC into the aorta under fluoroscopic guidance.

Many operators predilate the transcaval traversal site with the sheath dilator alone. Transient hypotension during exchange for the introducer sheath demonstrates how a specific patient will respond to acute shunting. This helps teams avoid overzealous blood transfusion or vasopressor administration in response to transient shunt-induced hypotension during later tract closure.

Next, the TAVR introducer sheath is advanced into the aorta under fluoroscopic guidance, ensuring that the sheath tip does not “flare” and injure the aortic wall (particularly if using an eSheath).

Nonuniformly expanding introducer sheaths (such as the eSheath) require a specific orientation during advancement from IVC to aorta, in order to prevent sheath tip flaring or even splitting. The cephalad aspect of the sheath experiences the most stress and deformation during transcaval advancement. Therefore, we recommend that the splitable seam of the eSheath be oriented to enter the aorta last, on the most caudad aspect of the aortocaval hole. This is accomplished simply by orienting the eSheath sidearm upward toward the ceiling during advancement. Note that the eSheath, if inadvertently withdrawn from the aorta, must be replaced rather than readvanced to prevent serious injury to the aortic wall. Take care also that the dilator does not advance beyond the aortic guidewire.

Ensure that the sheath has adequate purchase inside the aorta. Leave a guidewire inside the transcaval sheath at all times to protect against injury to the abdominal aortic wall during subsequent TAVR procedural steps and to help manage inadvertent sheath withdrawal. If sheath length allows, fixing the hub 2 to 3 cm from the skin may allow later tandem advancement of the transcatheter valve and sheath to address later difficulty. Flush and then suture the sheath securely to prevent dislodgement during TAVR. Aortography is not indicated at this point except in evaluation of unexplained hemodynamic instability.

Femoral transvenous pacemakers are best placed after the transcaval TAVR sheath, to avoid inadvertent advancement by the transcaval sheath. Balloon deflation sometimes helps the pacemaker to pass the transcaval sheath.

**FIGURE 7:**
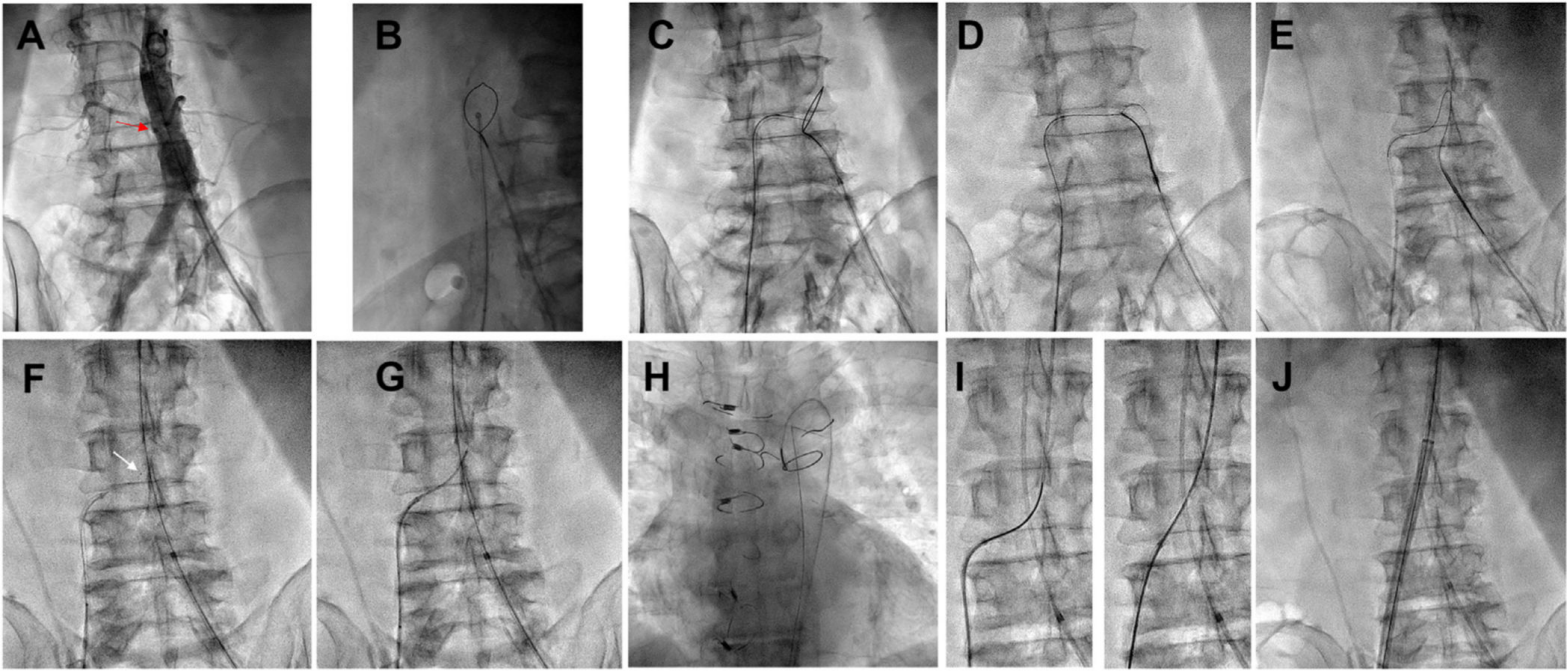
Traversal Sequence Representative traversal sequence. **(A)** Aortogram in computed tomography–derived projection. No venogram is required. **Arrow** indicates target. **(B)** Orthogonal projection confirms that the traversal system aims at the snare. **(C)** Electrified guidewire exits the inferior vena cava and enters the aorta. Electrification ceases when the wire crosses the snare border. **(D)** The aortic guidewire is ensnared while feeding forward to avoid outward traction on the aortic catheter. **(E)** The aortic guide is advanced, leading with a looped guidewire. **(F)** A 0.014-inch microcatheter **(arrow)** creates a 0.035-inch caval-aortic rail to deliver a 0.035-inch microcatheter **(G)**. **(H)** The 0.014-inch guidewire-microcatheters are released from the snare and withdrawn through the 0.035-inch microcatheter. **(I)** A Lunderquist-style 0.035-inch guidewire advances across the aortic wall **(left)** and straightens the microcatheter **(right)**. **(J)** The transcatheter aortic valve replacement introducer sheath advances into the aorta.

A representative crossing procedure sequence is shown in Figure 7.

The intended procedure (TAVR, TEVAR, MCS, etc) is then performed as usual.

**FIGURE 8:**
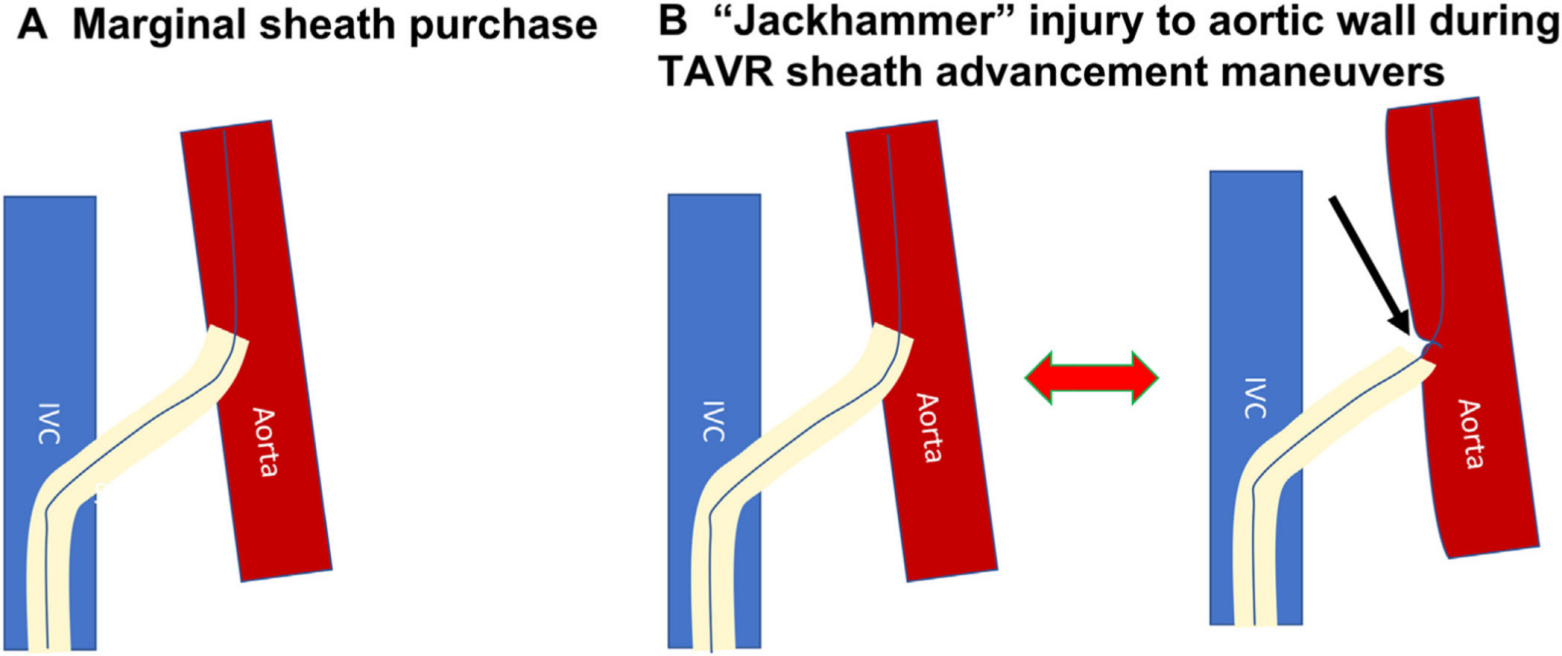
Jackhammer Injury **(A)** Borderline intra-aortic purchase of the transcaval transcatheter aortic valve replacement (TAVR) introducer sheath risks bleeding and aortic injury. **(B)** TAVR advancement against resistance may cause repeated “jackhammer” aortic injury. IVC = inferior vena cava.

Bear in mind that aggressive TAVR advancement force occasionally can push the transcaval introducer sheath out of the aorta. This may cause hypotension, especially because the sheath may simultaneously exit the aorta and obstruct blood return into the cava. Moreover, “jackhammer” exit and re-entry by the sheath tip against the aorta can injure the aortic wall and expand the hole (Figure 8). If the introducer sheath even partially exits the aorta during TAVR, the best solution is further to withdraw the sheath into the IVC, to restore the permissive aortocaval shunt physiology. A fresh sheath can be placed over the guidewire after TAVR is completed.

## CLOSURE PROCEDURES

The challenge of closure is to implant a wide-disk nitinol cardiac occluder device sideways to abut the aortic wall. Using available devices, which are permeable, the result is not usually immediate occlusion of the aortic hole but instead a tolerable aortocaval fistula that occludes over minutes to weeks.

**FIGURE 9:**
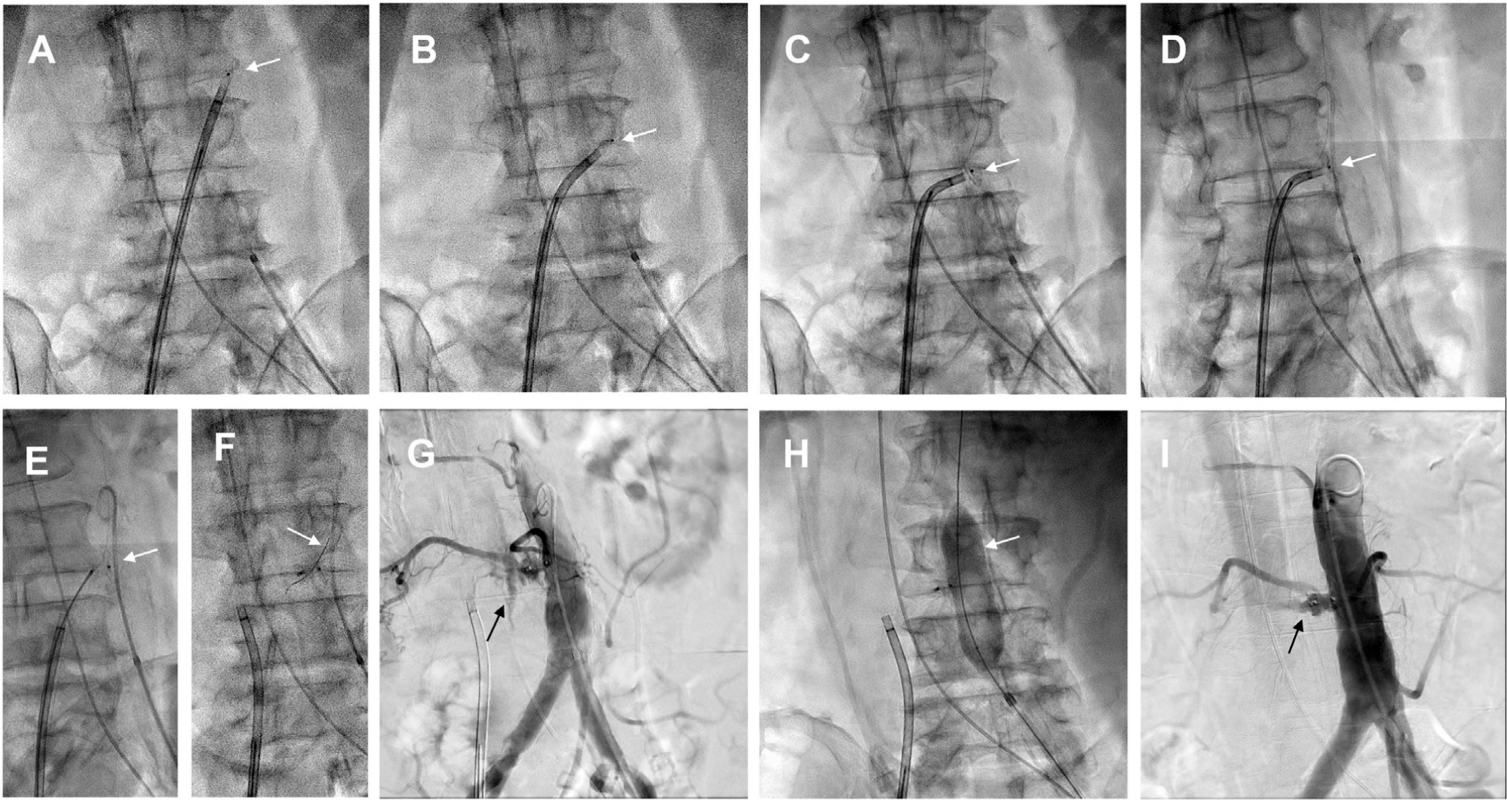
Closure Sequence Representative closure sequence. Protamine reverses heparin anticoagulation. **(A)** A 0.014-inch “buddy guidewire” across the aortocaval tract allows recrossing in case of inadvertent device pull-through. Partially exposed (“bulbed”) nitinol occluder. **(B)** A “bulbed” occluder turns sideways when withdrawn while deflecting the guiding sheath. **(C)** “Aortic” disc exposed sideways, but projection is not orthogonal. **(D)** Occluder withdrawn to abut the aorta. The right-left projection angle is adjusted until the disc appears tangential or orthogonal; its position along the aortic wall is unambiguous. **(E)** Occluder completely exposed while withdrawing the guiding sheath and advancing the delivery cable. **(F)** “Buddy” guidewire removed. **(G)** Device released from delivery cable. Hand contrast injection indicates extravasation. **(H)** Balloon aortic tamponade at low pressure for ≤3 cycles each up to 5 minutes. **(I)** Typical benign tubular residual aortocaval fistula without extravasation. The procedure is concluded.

Key closure steps are 1) assembling equipment and reversing anticoagulation; 2) implanting the closure device; and 3) reviewing arteriography, remedying complications, and concluding the procedure. Figure 9 illustrates a typical closure sequence.

### SPECIFIC EQUIPMENT AND MEDICATIONS. Protamine.

Heparin should be “fully reversed” using intravenous protamine before closure is initiated.^19^ The usual dose is 1 mg protamine for each 100 U heparin still circulating, assuming a half-life of 30 to 60 minutes. Rapid administration causes hypotension, so closure should be delayed a few minutes lest the cause of hypotension be uncertain. Excessive protamine does not appear thrombogenic.^20^

#### First-generation Amplatzer Duct Occluder.

**FIGURE 10:**
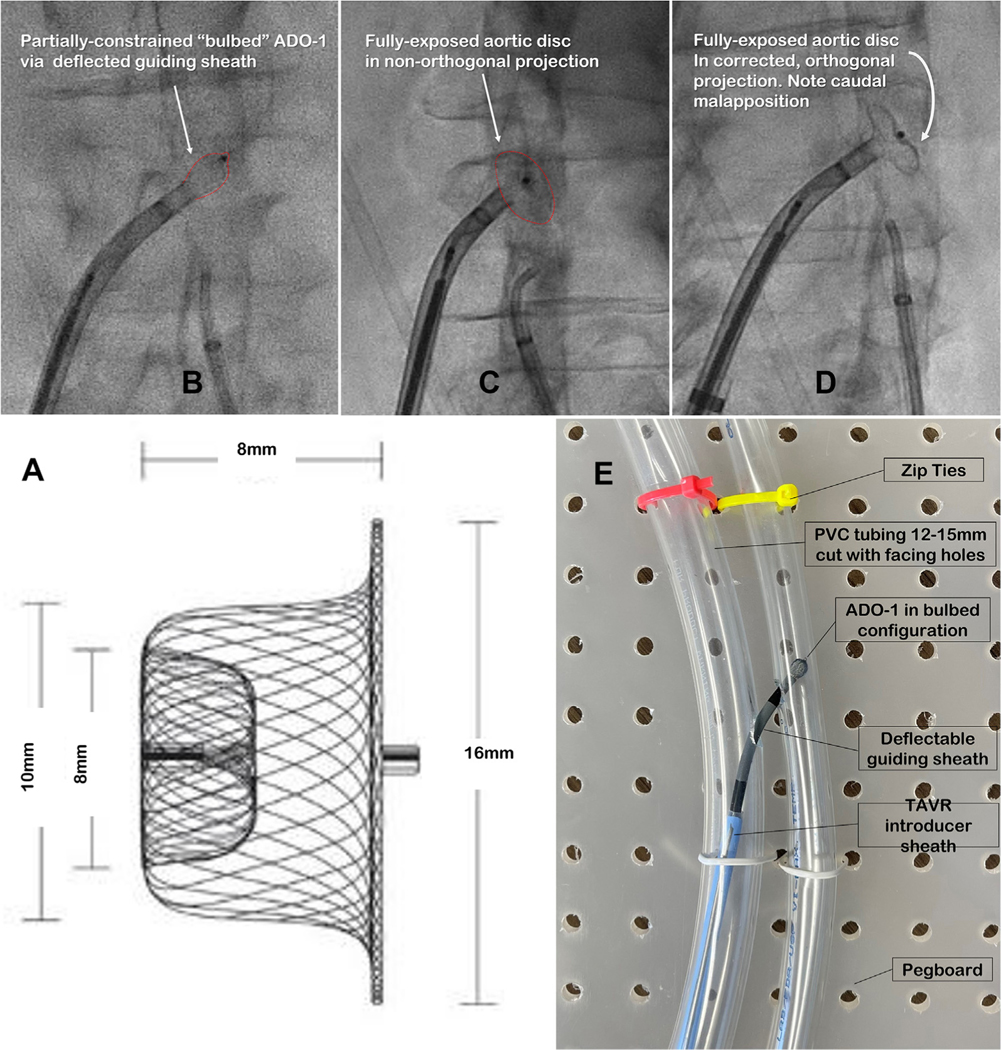
Bulbing Maneuver **(A)** A first-generation Amplatzer Duct Occluder (ADO-1; 9-PDA-006) tapers from the aortic wall toward the vein. Positioning is fixed by the 10-mm “neck,” while the 16-mm “disc” is more a visual aid. **(B)** A partial-exposure (“bulbing”) aortic disc while deflecting guiding sheath turns the ADO-1 sideways in small aortas. **(C)** The mural-luminal position of the exposed or retracted aortic disc unclear. **(D)** In the correct tangential or orthogonal projection, the aortic disc is unambiguously apposed to aortic wall. Note that caudal discs are typically malapposed. **(E)** Training phantoms using inexpensive parts: pegboard, clear polyvinyl chloride (PVC) tubing, and zip ties. Facing 6- to 8-mm holes cut in tubing recapitulate neighboring aortic and inferior vena cava holes. Simulated procedures use a deflectable guiding sheath, the ADO-1, and a delivery cable. TAVR = transcatheter aortic valve replacement.

For all TAVR devices used in the United States, which exhibit transient sheath outer diameter of at least 6 to 8.2 mm, we recommend the first-generation Amplatzer Duct Occluder (ADO-1; size 10/8, model 9-PDA-006, Abbott Vascular) (Figure 10A). The device has an aortic disc (“retention skirt”) diameter of 16 mm and an unconstrained neck diameter of 8 to 10 mm. It is screwed to a delivery cable and released by 6 counterclockwise turns. The Amplatzer delivery system is not used, except for the accompanying cable and loader (which lengthens the nitinol occluder so it can fit into a catheter).

Double-disk ventricular septal defect occluders add little value over the ADO-1. A purpose-built transcaval closure device exhibited favorable usability and hemostasis in human testing^21^ but awaits industry investment for commercial availability.

#### Deflectable guiding sheath.

Turning the closure device sideways for deployment can be challenging, especially in small aortas. We therefore recommend that a deflectable guiding sheath be used for all cases (such as the Nagare, Agilis [Abbott], or Direx [Boston Scientific]). Most have an inner diameter of 8.5 to 8.8 F, a usable length of 71 to 74 cm, and a total length of about 90 cm. We recommend the shortest available radius of deflection for all.

#### Emergency exchange devices.

The following accessory devices must be open and available:

- A soft 0.014 inch × 300 cm “buddy” guidewire to maintain transcaval access in case of accidental pull-through of the closure device during deployment. Stiffer buddy guidewires risk aortic laceration.
- The stiff 0.035-inch Lunderquist guidewire used earlier to place the transcaval introducer sheath.
- A commodity 0.035-inch J-wire to reposition the pigtail angiographic catheter during closure, if needed.
- A soft 4- to 5-F multipurpose diagnostic catheter to exchange the 0.014-inch buddy guidewire for the Lunderquist guidewire; alternatively the 0.035-inch microcatheter (eg, NaviCross) used for traversal can be reused here.
- A replacement TAVR introducer sheath (if an expandable TAVR sheath was used).

### IMPLANTING THE NITINOL OCCLUDER DEVICE.

Teams may benefit from a brief time out before tract closure, ensuring full heparin reversal, confirming availability of appropriate bailout equipment, and a reminder about shunt-related hypotension and “pseudo-hypotension” (described later) Table 6 summarizes key teaching points for closure.

The frontal fluoroscopic projection should be set to the same angle used for transcaval entry. The soft 0.014 inch × 300 cm “buddy” wire should be inserted through the TAVR sheath and advanced to the aortic arch. The TAVR guidewire should not be removed from the transcaval sheath but should instead first be exchanged for the deflectable guiding sheath into the abdominal aorta to prevent dissection. The transfemoral TAVR pigtail catheter should be withdrawn to just below the transcaval sheath, where it enters the aorta and connected to the injector manifold.

Next the suture retaining the TAVR introducer sheath is cut, and the nitinol occluder device is inserted into the deflectable guiding sheath. Amputating the distal Luer portion of the Amplatzer loader helps insert the nitinol occluder through the hemostatic valve into the deflectable guiding sheath.

The nitinol occluder is advanced to the tip of the deflectable guiding sheath until the aortic disc is partially exposed, in a maneuver we call “bulbing” (Figures 10B–10D). The bulbed disc allows the sheath to be maneuvered and deflected to turn the aortic disc sideways in an atraumatic way and without resistance, which is especially important in a narrow aorta.

The introducer is withdrawn swiftly out of the aorta, completely into the IVC, but preferably still within fluoroscopy window to ensure that it remains intravascular. Ensure that the sheath is withdrawn sufficiently that it does not block aortic blood return into the cava. Remind the staff that the blood pressure is expected to fall by about 20%.

The deflectable sheath and bulbed occluder are withdrawn to the vicinity of the aortic hole, which is also marked by the 0.014-inch buddy wire. The sheath is deflected approximately 90°. The aortic disc, now turned sideways, is fully exposed during iterative “push-pull” maneuvers as it is withdrawn to abut the aortic wall. Hand injections of contrast through the pigtail can be helpful. Transient aortic obstruction by the expanded nitinol occluder may cause pseudo-hypotension if pressure is transduced from the introducer sheath side arm.

Once the retracted aortic disc abuts the right wall of the aorta, the deflectable sheath is simultaneously straightened while it is withdrawn through the tract into the IVC and then further retracted to expose the entire nitinol occluder. The occluder delivery cable is pulled to ensure that the device reaches as far as possible into the cava and is then advanced to help the nitinol device reform (from its stretched to its thermal-memory shape) on the venous side. Typically, the shunt is largely relieved, and the blood pressure returns to baseline.

Because the aortic traversal point may not be at the location closest to the IVC, the aortic disc may land tangentially to the projection angle used for the original traversal. This can obscure significant malpositioning (Figure 10C). Therefore, the fluoroscopic projection should be adjusted to tangential to the aortic disc when it contacts the aortic wall, before aortography. The caudal aspect of the aortic disc is usually significantly malapposed without clinical sequelae (Figure 10D).

Importantly, the nitinol occluder is anchored by the neck against the aortic hole, while the aortic disk serves mostly to help position the device. Accordingly, the occluder cannot be repositioned without recapturing the device neck, which is a straightforward maneuver. However, device manipulation risks enlarging the aortic hole and should be minimized. Attempting to reposition ADO-1 devices using inflated balloons in the aorta is risky and rarely effective.

The pigtail catheter should not be entrapped by the nitinol occluder and, once the occluder is deployed, should be repositioned immediately cephalad to the occluder using the 0.035-inch J-guidewire if needed.

Digital subtraction aortography is performed in the updated projection angle, typically injecting 10 to 15 mL contrast over 1 second during a breath hold, with prolonged imaging (5 to 10 seconds) to detect subtle extravasation.

#### Inadvertent occluder pull-through during deployment.

The ADO-1 device is designed to lengthen under traction. Excessive traction can lead to inadvertent pull-through and unpredictably may enlarge the aortic hole. Fortunately, transcaval physiology usually maintains hemodynamic stability and allows comfortable remedy.

A soft 4- to 5-F multipurpose diagnostic catheter is advanced over the 0.014 inch × 300 cm transcaval buddy guidewire into the aorta. This should be performed under fluoroscopic guidance, lest the catheter fail to track into the aorta or, worse, prolapse the guidewire out of the aorta. Through the multipurpose catheter, the Lunderquist guidewire is advanced and can be used to deliver a replacement TAVR sheath or, if hemodynamic status allows, just the deflectable guiding sheath. An expanded eSheath should never be readvanced across the caval-aortic tract, as this is likely to cause serious aortic injury. Closure is conducted as described earlier.

It may be desirable to up-size the duct occluder to the 12/10-mm device (Amplatzer Duct Occluder, 9-PDA-007, Abbott) in case the aortic hole has enlarged.

Rarely, operators inadvertently withdraw all devices from the aortocaval tract before closure. The tract can be recrossed from the venous side retrograde using the deflectable guiding sheath or a cobrastyle catheter and a 0.014-inch coronary guidewire. An aortic pigtail catheter can serve as the crossing target.

#### Hypotension differential diagnosis.

Hypotension during closure is typically caused by one of the following: complications of TAVR; too rapid protamine infusion; bleeding; and aortocaval shunt, especially in the setting of biventricular failure and/or severe pulmonary hypertension.

### REVIEWING THE CLOSURE AORTOGRAM.

**FIGURE 11:**
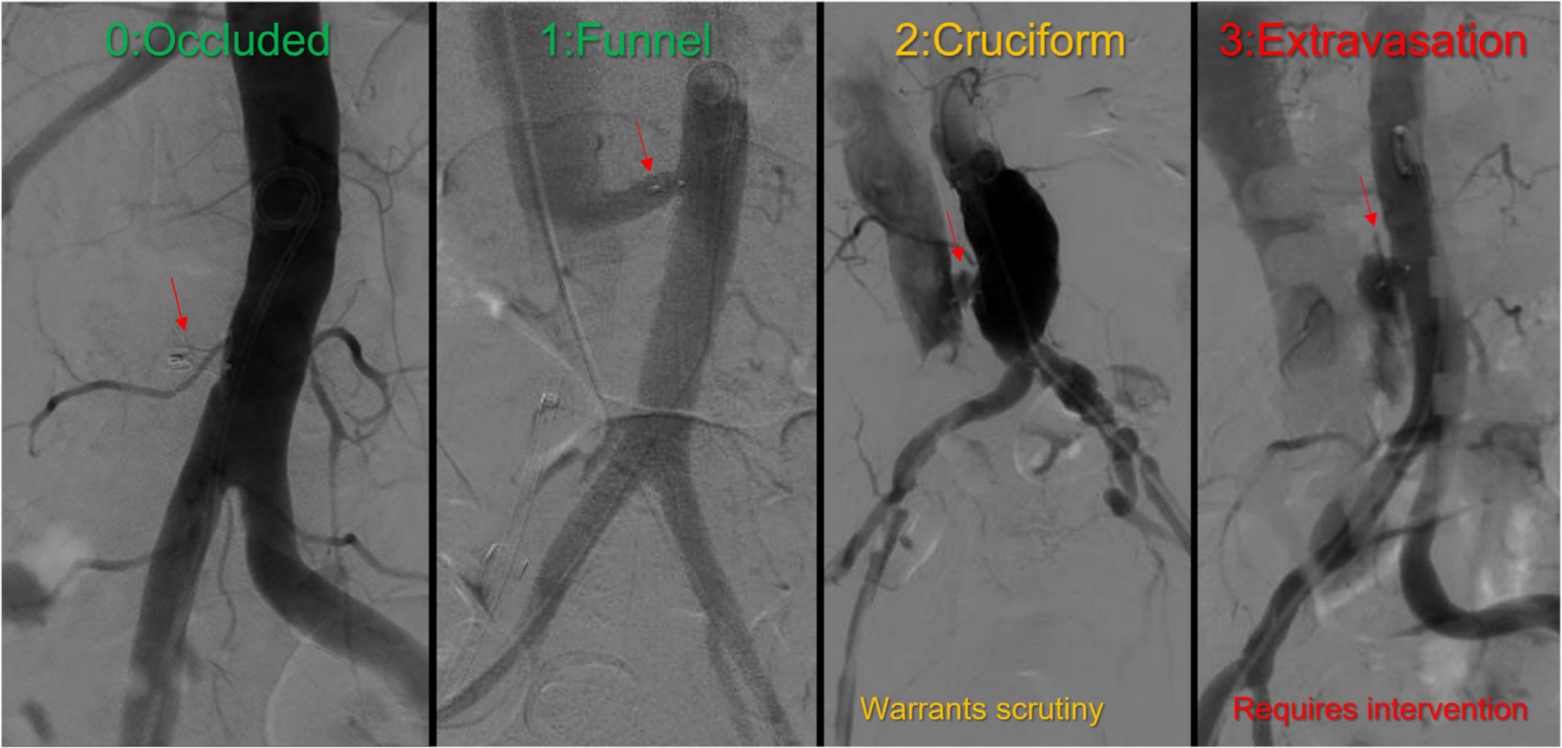
Closure Patterns on Completion Aortography Patterns of completion aortograms: complete occlusion (“type 0”) is now about 50% of cases. Funnel (“type 1”) and cruciform (“type 2”) patterns are functionally equivalent and reflect laminar (funnel) or vorticial (cruciform) blood flow. Cruciform patterns require digital subtraction angiography to ensure no extravasation (“type 3”) warranting immediate treatment.

Bowel gas may cause subtraction artifacts, so the aortogram should be evaluated carefully with and without subtraction and in comparison with the baseline digital subtraction aortogram. Figure 11 depicts typical aortography patterns after transcaval tract closure. In most contemporary cases with liberal use of protamine, the tract is occluded (“type 0 closure”). In the remainder, the aortogram shows a “funnel-shaped” (type 1) or “cruciform” (type 2) pattern, which are functionally equivalent. The latter is caused by contrast swirling around the free space between aorta and cava, constrained if at all by clot and interstitial fluid. The only important (but uncommon) finding is aortic extravasation, which requires immediate remedy (see the following section). Additional aortography projections can be helpful when the findings are ambiguous.

With the device implanted in the correct position, there are no other procedural steps involving the venous system. Any further diagnostic and therapeutic maneuvers will be confined to the arterial side. We therefore recommend withdrawing the 0.014-inch buddy guidewire, detaching the nitinol delivery cable (6 counterclockwise turns), withdrawing the caval catheters, and closing the femoral vein access site.

### REMEDIES.

If aortic extravasation is suspected or evident, a rigid 0.035-inch guidewire should immediately be exchanged for the aortic pigtail catheter, and a suitable introducer sheath should be placed to allow an aortic tamponade balloon to be positioned and inflated. Transducing the femoral artery sheath sidearm is very helpful to titrate balloon inflation volume to achieve tamponade without aortic injury. Fluoroscopy is helpful to ensure that the inflated balloon is positioned appropriately and not displaced caudally. We recommend 3 consecutive balloon cycles lasting 5 minutes, interrupted by 30-second deflations to allow distal perfusion. These steps can be performed after protamine and without administering additional heparin. Some operators perform at least 1 cycle of balloon aortic tamponade in all cases, analogous to routine femoral artery balloon tamponade. Table 7 lists large low-profile balloon occlusion or molding balloons available from several manufacturers; for aortas smaller than 13 mm in diameter, we use angioplasty balloon catheters that are compatible with sheaths ≤7F.

Afterward, another digital subtraction aortogram is obtained.

In the evolution of transcaval fistula thrombosis, rarely the venous side occludes before the aortic side. This should always be treated to prevent extravasation without venous return and usually responds to additional balloon aortic tamponade.

If extravasation continues, covered stent implantation is indicated. Table 8 lists specific covered stents used successfully for transcaval bailout in the United States and elsewhere, without excessive through-graft (type IV) endoleak. One specific device, the balloon-expandable Viabahn VBX (W.L. Gore), can be implanted beyond 11 mm nominal diameter into a 14-mm-diameter aorta in the presence of a transcaval Amplatzer closure device.

When the patient has hemodynamic instability related to the TAVR or other host conditions, combined with residual aortocaval fistula that might confound team management, we recommend a low threshold for placing a “prophylactic” covered aortic stent, assuming it can be deployed with little risk. This approach removes any ambiguity that transcaval shunting or bleeding might be contributing to postprocedural instability.

## SPECIAL SITUATIONS

### TRANSCAVAL ACCESS WITHOUT A CT PLAN.

Under exigent circumstances, transcaval access can be obtained without CT planning. Typically, this is for emergency percutaneous MCS device implantation, for which the risk tolerance is higher.

The average traversal level is the middle of the L3 lumbar vertebra, which is midway between the renal arteries and aortoiliac bifurcation. Cine fluoroscopy, during gantry rotation or exaggerated respiration, can help identify aortic wall calcification.

**FIGURE 12:**
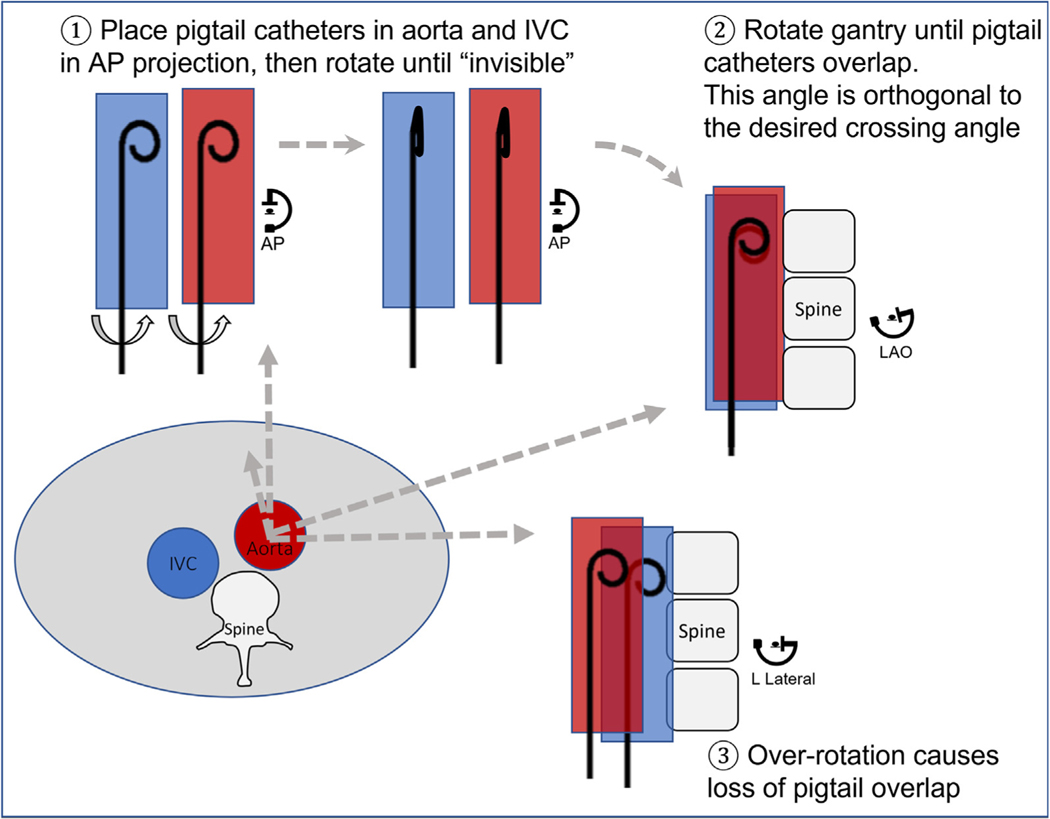
Finding Emergency Transcaval Access Projection Angles Without CT Finding optimal projection angles without computed tomography. The abdominal cross section depicts typical inferior vena cava (IVC), aorta, and spine relationships. **(1)** Pigtail catheters in the IVC and aorta are rotated in an anteroposterior projection until curves are no longer visible. **(2)** The fluoroscopy gantry is rotated left-right until pigtail catheters overlap. **(3)** Overrotation causes loss of catheter overlap.

Paired IVC and aortic pigtail catheters can be used quickly to identify optimal projection angles for transcaval access. With the pigtails rotated to have minimal diameter in the frontal projection, the radiographic gantry is rotated laterally until the 2 pigtails overlap. The best ad hoc crossing projection is orthogonal to this gantry position (Figure 12).

### PROLONGEDTRANSCAVAL ACCESS FOR PERCUTANEOUS MCS.

Transcaval access is an attractive alternative to transfemoral for indwelling MCS support, especially expected to last more than 2 days.^8^ It allows percutaneous implantation of the larger 5.0 L/min Impella (Abiomed) devices otherwise indicated for surgical implantation. When used in this fashion, the transcaval sheath should be flushed continuously to prevent thrombus formation in the large dead space around the smaller shaft of the MCS device.

Prolonged sheath implantation appears to reduce aortic mural recoil. We therefore recommend “upsizing” the nitinol occluder device, typically to a 12/10 ADO-1 (Amplatzer Duct Occluder, 9-PDA-007). In addition, we recommend removing and replacing the transcaval introducer sheath before advancing closure equipment as a secondary maneuver to prevent thromboembolism.

### PERCUTANEOUS CORONARY INTERVENTION, COAGULOPATHY, AND CIRRHOSIS.

Successful closure requires functioning host coagulation. Before undertaking percutaneous coronary intervention supported by transcaval MCS, operators should plan full anti-thrombin reversal before undertaking closure.

Similarly, coagulopathies especially related to impaired hepatic synthesis should be corrected, for example, with plasma transfusion before undertaking closure.

### RIGHT HEART FAILURE, SEVERE TRICUSPID REGURGITATION, INTOLERABLE SHUNT.

Elevated venous pressures induce correspondingly elevated interstitial pressure. Transcaval access and closure are successful in this setting.

However (as described earlier under “Hypotension Differential Diagnosis”), patients with severe right ventricular dysfunction or high pulmonary vascular resistance often do not tolerate even small acute left-to-right shunts, such as those for hemodialysis fistulae or for transcaval access. In such patients, persistent aortocaval fistula should be treated by balloon aortic tamponade or covered stent implantation.

### UNSUCCESSFUL AND ABANDONED TRAVERSAL ATTEMPTS.

Multiple failed (“abandoned”) attempts at transcaval guidewire entry to the aorta warrants special attention. Sometimes these create pinhole leaks that, if not adequately decompressed, can contribute to postprocedural bleeding. We recommend that repeated traversal attempts remain within millimeters of the abandoned hole, so that the large aortocaval fistula encompasses the earlier abandoned spot, and the nitinol closure device facilitates thrombosis and closure.

### MODERATE SEDATION.

General anesthesia is not required for transcaval access. That said, electrosurgical transcaval crossing can be painful. Awake patients should receive additional intravenous analgesia and/or sedation immediately before crossing. In addition to discomfort, patients “jumping” during traversal can cause slit-type injury to the aortic wall.

### TRANSCAVAL ACCESS WITHOUT A BAILOUT COVERED STENT OPTION.

Bailout covered stents are used in 1% to 5% of transcaval TAVR procedures. In a minority of patients, there is no suitably large device or access route to deliver a bailout covered aortic stent. Absent other good large-bore aortic access options, some well-informed patients, families, and physicians may choose transcaval access nevertheless, relying on careful management of heparin reversal and liberal application of balloon aortic tamponade.

### TRANSCAVAL ACCESS WITHOUT BAILOUT ILIOFEMORAL ARTERY ACCESS.

Percutaneous brachial or axillary access has been used for bailout covered stent placement in small aortas when there is no patent iliofemoral artery access. These have used smallcaliber covered stents premounted on 135-cm catheters (eg, Viabahn VBX).

### ABDOMINAL AORTIC GRAFTS.

As mentioned earlier, transcaval access is feasible across fabric surgical grafts.^17,18^

Two steps may require special attention. Operators may encounter difficulty delivering the 0.014-inch or 0.035-inch microcatheter, which may require ultralow-profile microcatheters or exchanging for extra-stiff 0.014-inch guidewires (such as the Platinum Plus, Boston Scientific) to accomplish balloon dilatation or laser atherectomy.

Balloon predilatation and/or sequential predilatation with oversized sheath dilators may be required to cross with the TAVR introducer sheath.

### PRIMARY CLOSURE WITH A COVERED STENT.

Deploying the ADO-1 nitinol occluder (with a 16-mm aortic disc) is difficult in patients with aortic diameters <10 to 12 mm. In this setting, some operators have chosen primary closure with a transfemoral covered stent, as listed in Table 8.

### INTERPOSED BOWEL.

In some especially elderly patients, CT imaging may show ptotic duodenum draped between the aorta and cava near the intended transcaval trajectory. Some experienced operators undertake successful transcaval access in this setting by 1) aiming for the posterior aspect of the aorta to avoid the bowel; and 2) using 2-step electrification to cause reflex visceral retraction.

### VENA CAVA FILTER.

IVC filters are a “soft” contraindication. Transcaval TAVR has been performed below, above, and alongside nitinol vena cava filters.

### SHEATHLESS TRANSCAVAL.

Transcaval TAVR has been performed without a TAVR introducer sheath using an Evolut R transcatheter heart valve (Medtronic)^18^ ad hoc when available introducers sheaths were found to be too short to reach the transcaval target. Such procedures may rely on oversized caval holes to allow continuous decompression of aortic bleeding. In general, we recommend against this strategy except as a bailout contingency.

## CONVALESCENCE AND COMPLICATIONS

Recovery after transcaval closure is similar to that after other large-bore access. Generous parenteral fluid administration compensates for shunt-related volume shifts.

If there is persistent hypotension despite fluid resuscitation in the recovery unit, without evident TAVR-related complication, noncontrast CT imaging is helpful to exclude retroperitoneal hemorrhage. Contrast-enhanced CT imaging is not recommended, because of the additional iodinated contrast load. If retroperitoneal hemorrhage is observed on noncontrast CT imaging and hypotension persists, the patient should return to the catheterization laboratory for angiography and further intervention. A “take-back” angiogram can also demonstrate and allow treatment for the uncommon circumstance of intolerable fistula, typically in the setting of right ventricular failure and/or severe pulmonary artery hypertension.

We no longer recommend routine surveillance contrast CT imaging after transcaval access and closure. Almost all residual caval-aortic fistulae spontaneously close over hours to months. In systematic follow-up, 1% of fistulae remained patent at 12 months, and there were no subacute or late transcaval vascular complications or occluder fracture or migration.^3^

In general, surgical bailout is not indicated after transcaval access. Surgical exposure of an otherwise intact retroperitoneal space may exacerbate bleeding. No surgical venous interventions are indicated. And there are few circumstances in which a preplanned covered aortic stent does not satisfactorily mitigate bleeding.

## TRAINING IN TRANSCAVAL ACCESS

Inexperienced operators can assemble an inexpensive phantom to learn the required skills. Our homemade phantom has 2 clear vinyl 12-mm-diameter tubes ziptied 1 to 2 cm apart on a wooden pegboard (Figure 10E), with two 8- to 10-mm circular holes cut on opposing sides to model an aortocaval tract. Operators can practice “bulbing” and related maneuvers to deploy the nitinol occluder across this tract.

We recommend that an experienced transcaval operator proctor first transcaval procedures. Proficiency typically requires experience with 2 procedures.

## CONCLUSION

With meticulous CT planning and by following the technical steps described in this review, transcaval access offers a safe, nonfemoral access route for TAVR and for other interventions that require large-bore arterial access.

## Figures and Tables

**CENTRAL ILLUSTRATION:**
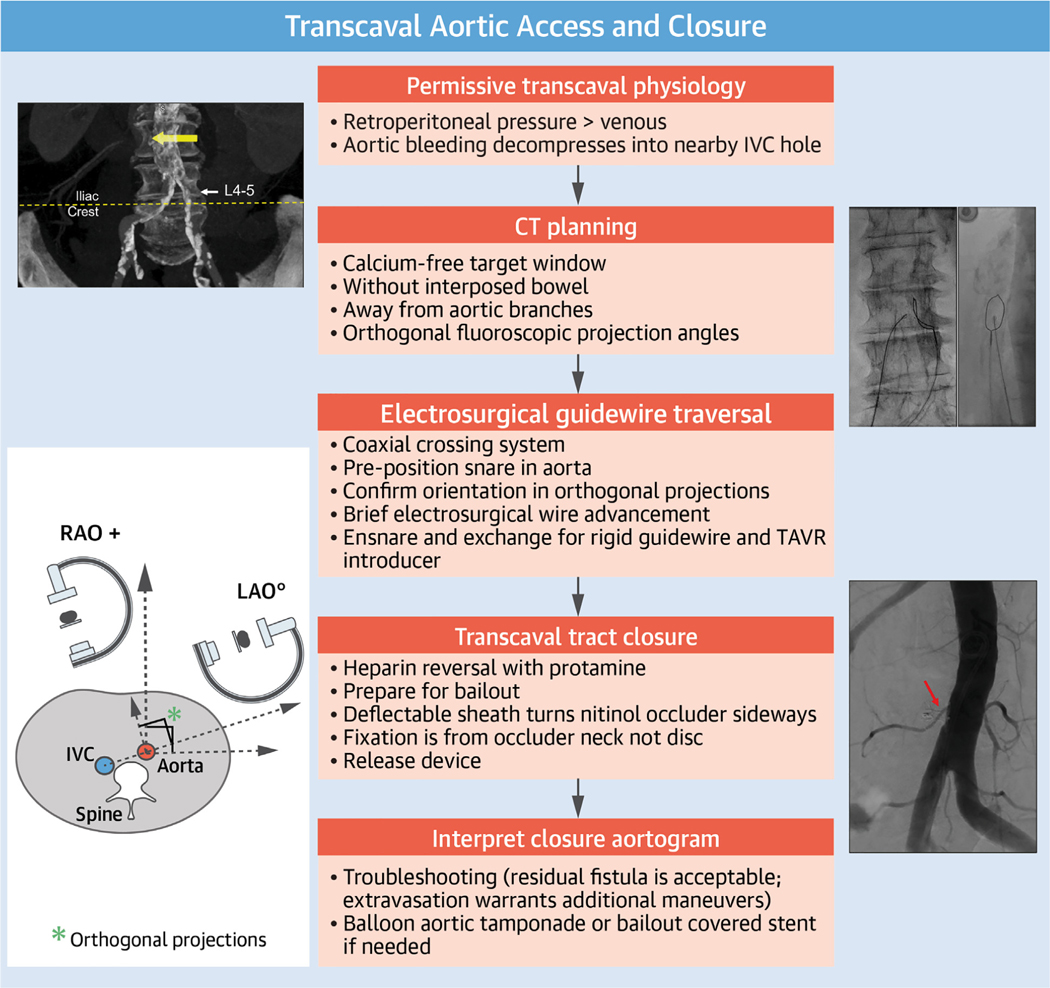
Transcaval Aortic Access CT = computed tomographic; IVC = inferior vena cava; LAO = left anterior oblique; RAO = right anterior oblique; TAVR = transcatheter aortic valve replacement.

**TABLE 1:** Computed Tomographic Planning

**Key teaching points for computed tomography-based transcaval planning**	
• Key features of transcaval targets ∘ Having a calcium-free window on the caval side of the aorta ∘ Located away from important arterial branches ∘ Have no interposed viscera or right renal artery ∘ Have a femoral vein-to-transcaval distance suitable for the length of the intended introducer sheath • Caval-aortic distance does not matter • Calcium-free targets are most quickly found on volume rendering viewed from the IVC • Interposed venous structures are common and unimportant • Check for celiac and superior mesenteric artery patency • Aim for targets on the caudal aspect of calcium-free windows • Leftward aorta segments ≥30° are unattractive targets	
**Computed tomographic plan checklist**	
Parameter	Measurement
• Intended vascular introducer sheath	
∘ Outer diameter (including valve)	_____ mm
∘ Working length	_____ cm
• Distance from lowest renal artery	_____ mm
• Distance from aortoiliac bifurcation	_____ mm
• Target calcium-free window dimension	_____ mm
• Caval-aortic distance	Irrelevant
Interposed vulnerable structures	Bowel: Yes/No
	Renal artery branch: Yes/No
Estimated groin-to-transcaval distance (recommended 3–5 cm shorter than sheath working length	_____ cm
Projection angle	Anterior _____ degrees RAO/LAO
	Lateral _____ degrees RAO/LAO
Mesenteric arteries	Celiac patent: Yes/No
	SMA patent: Yes/No
Aortic diameter	15 mm above target: _____ mm
	Target: _____ mm
	15 mm below target: _____ mm
Unsuitable targets	Aortic dissection: Yes/No
	Pedunculated atheromata: Yes/No

IVC = inferior vena cava; LAO = left anterior oblique; RAO = right anterior oblique; SMA = superior mesenteric artery.

**TABLE 2:** Equipment List

Category	Device	Part Number	Per Case	Note
Access	Perclose ProGlide	12673-03	10	Venous access site preclose: can use “figure-of-8” stitch as alternative.
Target	Amplatz Goose Neck snare (Medtronic) ONE Snare (Merit)	15 mm: GN1500	1	For guidewire target and capture. Sized 5 mm larger (rounding up) than aortic luminal diameter at target site, typically use 20 or 25 mm (stock 15, 20, 25, and 30 mm). *Do not use multiloop designs*. Used inside a guiding catheter + locking hemostatic valve + stopcock.
		20 mm: GN2000		
		25 mm: GN2500		
		30 mm: GN3000		
		15 mm: ONE1500		
		20 mm: ONE2000		
		25 mm: ONE2500		
		30 mm: ONE3000		
Target	Guiding catheter, JR4, 6-F coronary length (90–100 cm)	6 or 7-F any	1	Used in tandem with Goose Neck snare instead of supplied catheter to aid in positioning of snare, Tuohy + stopcock.
Crossing	Cordis RDC, RDC-1, IM, JR4 guiding catheter, renal length (55 cm), 6, 7 or 8-F	778-212-55 778-210-55 G780JR4D	1	Directs crossing system: • IMA to direct horizontally • RDC-1 for narrower IVC < ~20–25 mm • RDC for >25 mm • JR4 or IMA for <20-mm IVC + Tuohy + stopcock—Copilot preferred (allows easy movement of braided microcatheter)
Crossing	PiggyBack wire convertor, 145 cm (Vascular Solutions)	8802	2	Premounted over the AstatoXS20. Strongly preferred over alternative 0.014-inch microcatheters Stock extras in case of kinking.
Crossing	Astato XS 20 (Asahi), 300 cm	PAGH143392	1	Preloaded inside PiggyBack microcatheter.
Crossing	Braided microcatheter, straight tip, 90 cm length, 0.035-inch ID	• NaviCross (Terumo): NC35900 • Minnie (Vascular Solutions): 5706 • Quick-Cross Extreme (Spectranetics): 518-078 • Seeker (Bard): SK9035M	1	Preloaded with Astato inside PiggyBack inside braided microcatheter: 0.035-inch tested alternatives are listed.
Crossing	Alternative microcatheter example: Terumo FineCross MG	• Terumo FineCross MG 130 cm (35–1430) or 150 cm (35–1450)	1	Braided microcatheter alternative to PiggyBack; use in combination with NaviCross.
Crossing	Needle driver, large	Sklar 96-2539	1	Or equivalent. For clamping to Bovie.
Crossing	Electrosurgical pencil “Bovie” system	Covidien E2450H	1	Or equivalent. With patient-indifferent electrode (ground pad) and electrosurgery system: set at 50 cutting to start.
Crossing	Lunderquist Extra-Stiff guidewire, straight, 0.035-inch × 260 cm (Cook)	TSCMG-35-260-LES (G45353)	1	Or equivalent: for sheath delivery and for emergency sheath redelivery. Note that the double curve may “pull” the microcatheter out of the aorta during exchange, so we recommend the straight or single-curve wires.
Crossing	Sheath: for Edwards SAPIEN 3 1) 14-, 16-, 18-, or 20-F eSheath 2) Cook large Check-Flo Performer for backup 3) Cook extra-large Check-Flo Performer (≥20-F)	• Edwards eSheath • Cook RCFW-18.OP-38-40-RB • Cook XVCFW-20.0-35-40 for backup	2 of each	*Do not use Terumo SoloPath*. Ensure sheath working length comfortably exceeds femoral-caval-aortic distance. Desirable working lengths are 35–40 cm. Retain sheath dilator for emergency recrossing (applies only to Cook sheaths). *Do not recross with a used eSheath*; if using eSheath, have immediately available in-lab Cook RCFW-18.OP-38-40-RB in case of need to recross.
	Sheath: for Medtronic Evolut valves Cook large Check-Flo Performer (18-F)	Cook RCFW-18.OP-38-40-RB	2	*Must be the 40-cm length*.
	Sheath: for Impella Gore DrySeal Flex	Gore DrySeal Flex DSF2233 22-F × 33 cm		33 cm working length; also available up to 26 REVIEWING THE CLOSURE AORTOGRAM.
Closure	Multipurpose diagnostic catheter 4-5-F, coronary length	Cordis Infiniti 534-542T (5F MPA2) or equivalent	1	Or equivalent. Bailout contingency, for emergency recrossing of buddy guidewire to exchange for Lunderquist. Have open on table.
Closure	0.014 inch × 300 cm medium guidewire	Any, such as BMW	1	Bailout contingency, buddyguidewire before deploying. Stiff wires are undesirable because they may lacerate.
Closure	Amplatzer Duct Occluder first generation 10/8, 12/10, and 8/6	9-PDA-006 9-PDA-007	1 each	For closing the access tract. Use only first generation. Upsize in case device is pulled through during closure or in case of protracted implantation (such as Impella >24 h). Do *not* use ADO2 (which pulls through too easily, insufficiently hemostatic).
Closure	Amplatzer TorqueVue Delivery Catheter, 45° (7, 8-F)	9-ITV07F45/60 9-ITV08F45/60	1	Contains required cable and loader used with deflectable guiding sheath. The 7-F size is preferred, and its distal Luer is amputated with scissors to help introduce into deflectable sheath.
Closure	AgiLis NxT SML curve 8.5-F × 71/91 cm, OD12-F DIREX sheath smaLL curve angled dilator, 8.9-F × 71/91 cm, OD12.3-F Nagare (Terumo) 8.8-F × 73.7/91.4 cm, OD 12-F, 0.032 inches	408309 (Abbott) M004 DS10 O (BSC, Oscor) SS8FMB74 (Terumo)		Preferred over Amplatzer delivery system (helps align aortic disc of occluder during pull back). Use the small (“SML”) curve.
Contingency	EndoLogix iLiac Limb extender covered stents and deLivery system	I16-16/C55:16 × 55 mm iLiac Limb I20-20/C55: 20 × 55 mm iLiac Limb A25-25/C55: 25 × 55 mm aortic extension A28-28/C55: 28 × 55 mm aortic extension	1 +	These work best compared with Cook, Gore, Medtronic; outer graft is hemostatic, self-expanding nitinol is conforming, 16- and 20-mm diameter grafts can be delivered “bare” without delivery system. The 20 × 55mm iliac limb extension is the most common size.
Contingency	TrivascuLar (an EndoLogix company) Ovation iX iLiac Limb extensions	16,18, 22 and 28 mm (diameter) x 45 mm (Length)	≥1	The 16- and 18-mm extensions are alternatives to the 16- and 20-mm Endologix extensions with slightly smaller delivery sheath profiles; the 22- and 28-mm extensions are particularly beneficial, as they do not require any integrated delivery system and have significantly smaller delivery profiles than the 25- and 28-mm Endologix extensions.
Contingency	Gore VBX	11 mm x 39 or 59 mm BXA113902A BXA115902A		Supplied on 11-mm delivery balloon. Can be postdilated up to 16-mm diameter. Shortens. Have appropriate postdilatation balloons available.
Contingency	PTS Sizing BaLLoon (B. Braun) 8/9-F × 30/40 mm Tyshak-II (B. Braun) 9/10-F × 25/30 mm MoLding & OccLusion BaLLoon (Gore) 10-F × 37 mm Q50X (Merit) 10-F × 50 mm Aortic BaLLoon (Terumo) 10-F × 50 mm ReLiant (Medtronic) 12-F × 50 mm Coda (Cook) 12-F × 32 mm	B. Braun: 613703 (30 × 4); 613706 (40 × 4) B. Braun: 611944 (25 × 4) Gore: MOB37 Merit: Q50 PLus Q50-65P Medtronic: REL46 Cook G03832 CODA-2-9.0-35-100-32	1	For emergency temporary occlusion of the aorta. Compatible with 12-F sheath. Can be used for prolonged inflation.
Contingency	Introducer sheath for ReLiant or Coda 12-F × short	Any brand 12-F × 10–14 cm	1	This needs to be in the room for all cases for emergency balloon aortic tamponade.
Contingency	Armada 035 PTA Catheter (Abbot)	B1140-040:14 mm × 4 cm × 80 cm B1120-020:12 mm × 4 cm × 80 cm	1 each	Or equivalent; 7-F compatible balloon catheters for very low pressure inflation. Oversize to nominal aortic target caliber for adjunctive aortic PTA or for emergency aortic occlusion

Recommended equipment list for transcaval transcatheter aortic valve replacement. *Do not substitute* except as noted. These recommendations should not be construed as product endorsements. ADO2 = second-generation Amplatzer Duct Occluder; ID = inside diameter; IM = internal mammary; IMA = internal mammary artery; IVC = inferior vena cava; OD = outside diameter; PTA = percutaneous transluminal angioplasty; RDC = renal double curve.

**TABLE 3:** Checklist for Preprocedural Staff Briefing

• Anesthesia and sedation staff
∘ If using moderate sedation, consider giving extra analgesia before traversal
∘ Do not overreact to hypotensive effect of acute shunt
∘ Avoid overzealous blood transfusion and vasopressor administration
• Technologist and nursing staff
∘ Review the patient-specific CT-based plan
∘ Assemble bailout equipment inside the procedure room: balloon aortic tamponade catheter and introducer sheath, suitable covered stent system
∘ Configure the electrosurgical generator: monopolar “pure” cutting mode at 30–50 W, with dispersive electrode properly applied
∘ Scrape insulation from back end of electrosurgery wires used off label
∘ Minimize flame hazard: avoid an oxygen-flooded field
∘ Full heparin administration immediately after vascular access is obtained and before traversal is attempted
∘ How to react to partial sheath withdrawal from aorta (by further withdrawing into cava)
∘ Full heparin reversal before undertaking closure

CT = computed tomography.

**TABLE 4:** Key Teaching Points for Crossing

• Select a guiding catheter to cross horizontally, orthogonal to aorta
• Full anticoagulation before crossing to prevent thromboembolism
• Ensure that the back end of the guidewire and the electrosurgical pencil are not insulated and have a good electrical connection
• Confirm fine positioning of crossing equipment in orthogonal (frontal and en face) fluoroscopic projections using the aortic snare as a bull’s-eye target
• Cross at the most caudal aspect calcium-free “windows” on the aorta
• Electrify only briefly, to prevent char and to enable vaporization rather than heating
• Do not allow electrified guidewires to buckle
• Buckling of nonelectrified wires on the contralateral aortic wall usually confirms aortic luminal entry
• When snare-locking (invaginating) transcaval guidewire in the aortic guiding catheter, ensure enough redundant guidewire to avoid forcing the aortic guiding catheter out of the transcaval tract
• Provide firm countertraction to allow serial exchange of microcatheters
• Use “dilator predilatation exchange” test to evaluate shunt hemodynamics before positioning the TAVR introducer sheath

TAVR = transcatheter aortic valve replacement.

**TABLE 5:** Common Causes of Electrosurgical Failure

• Dispersive electrode poorly connected to patient skin; apply well to clean and dry skin as well as to generator.
• Failure to denude the proximal tail of the guidewire where it connects to the electrosurgical pencil.
• Unrecognized use of an insulated (edge) electrosurgical pencil face. Replace with uninsulated electrosurgical pencil, or completely scrape away insulation.
• Carbonization (“char”) of the guidewire from prolonged prior electrosurgery, requiring scraping, amputation, or replacement of the guidewire tip. Guidewires energized >3 or 4 times should probably be removed, inspected, scraped, and/or replaced.
• Guidewire short circuited (eg, wet table drapes, not connected to electrosurgery system).
• Failure to activate the “cut” mode on the electrosurgical generator (pressing the “blue” electrocoagulation button rather than the “yellow” cutting button on the electrosurgery pencil).
• Inadequate energy setting (may need cutting mode set to 50–70 W or more).

**TABLE 6:** Key Teaching Points for Closure

• Fully reverse heparin with protamine.
• Always insert a buddy 0.014-inch guidewire alongside closure device.
• Amputate the Amplatzer loader to ease device advancement into the guiding sheath.
• “Bulb” the (large-disc) nitinol occluder to turn it sideways (in small aortas) using a deflectable guiding sheath.
• Do not obstruct venous outflow with the TAVR introducer during closure; pull it all the way back into the IVC.
• Optimize the tangential projection angle for aortography once the aortic disk is abuts the aortic wall.
• Fixation comes from device neck; position comes from aortic disk; do not reposition the device using aortic balloon dilatation.
• Do not overreact to transient shunt-related hypotension.
• Recognize “pseudo-hypotension” caused by exposed aortic discs obstructing aorta during positioning.
• Clinically significant venous bleeding has not been observed; the venous hole need not be considered for closure.
• Carefully examine the completion digital subtraction aortogram for subtle extravasation.

IVC = inferior vena cava; TAVR = transcatheter aortic valve replacement.

**TABLE 7:** Options for Balloon Tamponade of Aortas >13 mm in Diameter

Device	Manufacturer	Required Introducer, F	Maximum Diameter, mm
Amplatzer Sizing Balloon II	Abbott	6/7/8	20/27/40
PTS Sizing Balloon	B. Braun	8/9	30/40
Tyshak-II	B. Braun/Numec	9/10	25/30
Molding & Occlusion Balloon	Gore	10	37
Q50X	Merit	10	50
Aortic Balloon	Terumo	10	50
Reliant	Medtronic	12	50
Coda	Cook	12	32

**TABLE 8:** Bailout Covered Stent Options

United States			Everywhere Else		
Aorta Size, mm	Covered Stent	Device OD, F	Aorta Size, mm	Covered Stent	Device OD, F
9–14	Viabahn VBX (W.L. Gore) 11 × 59, 39, or 29 mm	8	9–14	Viabahn VBX (W.L. Gore) 11 × 59, 39, or 29 mm	8
14–18	AFX (Endologix) 16 × 55 mm 20 × 55 mm (delivered bare)	14	14–24	3eGraft Aortic (Bentley) 14 × 29, 39, 49, or 59 mm 16 × 29, 38, 48, or 58 mm	11
18–26	Ovation iX (Trivascular) 22 × 45 mm, 28 × 45 mm	14,15	24–30	3eGraft Aortic (Bentley) 20–24 × 27, 37, or 48 mm	14

OD = outside diameter.

## HIGHLIGHTS

- Transcaval access, while counterintuitive, is a straightforward percutaneous method.
- Transcaval access is helpful for TAVR and percutaneous MCS.
- Best practices address CT planning, electrosurgery, closure, and troubleshooting.
- Readers can adopt transcaval access as a core large-bore aortic access strategy.

## PERSPECTIVES

### WHAT IS KNOWN?

Transcaval access, seemingly counterintuitive, is a straightforward percutaneous large-bore aortic access technique. Transcaval is used for TAVR and to implant large-caliber mechanical circulatory support devices.

### WHAT IS NEW?

Absent industry sponsorship, this summary of best practices should help operators achieve or maintain proficiency in transcaval access including CT-based planning (calcium-free target selection, projection angles, bailout strategy), electrosurgical guidewire traversal, closure, and troubleshooting.

### WHAT IS NEXT?

Broader clinical exposure and dedicated tools will help operators to adopt transcaval access as a core non-femoral access approach.

## ABBREVIAT IONS AND ACRONYMS

ADO-1: first-generation Amplatzer Duct Occluder
CT: computed tomography
IVC: inferior vena cava
MCS: mechanical circulatory support
TAVR: transcatheter aortic valve replacement
TEVAR: transcatheter endovascular aortic repair
